# Genome-wide identification and analysis of WD40 proteins in wheat (*Triticum aestivum* L.)

**DOI:** 10.1186/s12864-018-5157-0

**Published:** 2018-11-06

**Authors:** Rui Hu, Jie Xiao, Ting Gu, Xiaofen Yu, Yang Zhang, Junli Chang, Guangxiao Yang, Guangyuan He

**Affiliations:** The Genetic Engineering International Cooperation Base of Chinese Ministry of Science and Technology, Key Laboratory of Molecular Biophysics of Chinese Ministry of Education, College of Life Science and Technology, Huazhong University of Science and Technology (HUST), Wuhan, 430074 China

**Keywords:** *Triticum aestivum*, WD40 proteins, Expression profiles, Seed development, Biotic and abiotic stresses

## Abstract

**Background:**

WD40 domains are abundant in eukaryotes, and they are essential subunits of large multiprotein complexes, which serve as scaffolds. WD40 proteins participate in various cellular processes, such as histone modification, transcription regulation, and signal transduction. WD40 proteins are regarded as crucial regulators of plant development processes. However, the systematic identification and analysis of WD40 proteins have yet to be reported in wheat.

**Results:**

In this study, a total of 743 WD40 proteins were identified in wheat, and they were grouped into 5 clusters and 11 subfamilies. Their gene structures, chromosomal locations, and evolutionary relationships were analyzed. Among them, 39 and 46 pairs of *TaWD40*s were distinguished as tandem duplication and segmental duplication genes. The 123 *OsWD40*s were identified to exhibit synteny with *TaWD40*s. TaWD40s showed the specific characteristics at the reproductive developmental stage, and numerous TaWD40s were involved in responses to stresses, including cold, heat, drought, and powdery mildew infection pathogen, based on the result of RNA-seq data analysis. The expression profiles of some *TaWD40*s in wheat seed development were confirmed through qRT-PCR technique.

**Conclusion:**

In this study, 743 *TaWD40*s were identified from the wheat genome. As the main driving force of evolution, duplication events were observed, and homologous recombination was another driving force of evolution. The expression profiles of *TaWD40*s revealed their importance for the growth and development of wheat and their response to biotic and abiotic stresses. Our study also provided important information for further functional characterization of some WD40 proteins in wheat.

**Electronic supplementary material:**

The online version of this article (10.1186/s12864-018-5157-0) contains supplementary material, which is available to authorized users.

## Background

WD40 proteins are also called WD40 domain-containing proteins, and they constitute a large gene family among eukaryote species [1]. WD40 is named from the WD dipeptide of its conserved domain and defined that every single repeat contains 44–60 amino acids, and there are a GH dipeptide and a WD dipeptide at N- and C-terminals, respectively [2]. Bovine β-transducin is the first identified WD40 protein [3], and its crystal structure possesses a seven-bladed β-propeller fold formed by repeats [4–6]. A strong hydrogen bond network produced by this WD domain structure improves the stability of WD40 proteins [7]. WD40 proteins exist extensively in all eukaryotes, but they are rarely found in prokaryotic proteomes, though a WD40 protein named PkwA was identified in a prokaryote in 1996 [8]. Only a small proportion of archaea proteomes (27 of 134) and bacterial proteomes (466 of 1679) have WD40 proteins [9].

The WD40 domain is slightly conservative in terms of protein sequence, thus, precisely predicting the number of WD40 repeats in a WD40 protein is difficult. The prevailing prediction methods for the analysis and identification of a WD40 domain are too conservative, for instance, the structural information of DNA damage binding protein 1 (DDB1) [10] and Sro7 [11] comprises seven or multiples of seven blades, but only some repeats can be identified through sequence-based classification methods in the prediction of their structures.

WD40 proteins are usually regarded as the scaffold of protein–protein interactions [12]. The number of WD40 repeats that form a β-propeller fold ranges from 5 to 8, more likely 7 [13, 14]. WD40 proteins have complex structures and functions; they can interact with diverse proteins in various ways and participate in extensive biological regulatory processes, such as DNA replication, damage response, histone recognition, transcriptional regulation, post-translational modification, signal transduction, protein degradation, and apoptosis [15–24].

The WD40 protein family has been systematically identified in plant species, humans, silkworms, and prokaryotes [9, 25, 26]. It is reported that there were 237 *WD40*s in thale cress (*Arabidopsis thaliana*), 191 *WD40*s in cucumber (*Cucumis sativus*), 225 *WD40*s in foxtail millet (*Setaria italica*), and 579 *WD40*s in cotton (*Gossypium hirsutum*), respectively. [27–30]. In the monocotyledonous model plant rice (*Oryza sativa*), 200 WD40 family members are clustered into 5 groups and classified into 11 subfamilies based on their domain compositions [31]. Although the WD40 family has been studied in many plant species, it has yet to be investigated in wheat (*Triticum aestivum* L.), which is an important cereal crop worldwide. Wheat is rich in proteins, carbohydrates, and minerals. According to the Food and Agriculture Organization (FAO), wheat production is predicted to account for 28.46% of the global cereal production (2.59 billion tons) by 2017/2018 [32]. Bread wheat can strongly adapt to different climates, and one of the key factors of this characteristic is the allohexaploid genome structure, which originates from two polyploidization events [33]. Allotetraploid *T. turgidum* (AABB), the ancestor of *T. turgidum* ssp. *durum*, is initially generated from the cross between *T. urartu* (AA) and *Aegilops speltoides* (SS) [34]. *T. turgidum* (AABB) subsequently crossed with *A. tauschii* (DD), and the ancestral allohexaploid *T. aestivum* (AABBDD) was finally obtained [33–39]. As a kind of allohexaploid plant species with 21 chromosomes, bread wheat has three homologous subgenomes (A, B, and D), and each subgenome contains seven chromosomes, but it genetically behaves like a diploid species [40]. The large and repetitive genome of wheat makes it very difficult to analyze the gene family on the basis of the wheat genome.

In this work, we identified TaWD40 proteins in wheat at a genome-wide level, including their number, chromosomal distribution, gene structure, gene duplication, and evolutionary relationship of family members. We analyzed the syntenic relationships of WD40 proteins between wheat and rice. We also examined the tissue-specific expression and expression profiles of *TaWD40*s under biotic and abiotic stresses by using the public RNA-seq data. Our qRT-PCR results indicated the possible important roles of some TaWD40s during seed development. All above-mentioned results provided a basis for further functional characterization of WD40 proteins in wheat.

## Results

### Identification of WD40 proteins in wheat

To identify the *TaWD40*s in wheat, the hmmsearch program (HMMER3.0 package) was performed against the protein databases (IWGSC RefSeq v1.0) by using the hidden Markov model (HMM) profiles of the WD40 domain (PF00400) as queries [41, 42]. After the redundant transcripts were removed, all of the candidates were examined via the HMMER website [43] and SMART website [44] to search for the WD40 domain. All of the published TaWD40 proteins on NCBI were screened, and these proteins were included in our result. A total of 743 WD40 proteins were obtained in wheat. For convenience, their corresponding genes were numbered from *TaWD40–1* to *TaWD40–743* based on their positions located on 21 chromosomes (Additional file 1: Table S1), from the top to the bottom, from 1 to 7, and in the order of A, B, and D [45].

In silico analysis revealed that TaWD40s varied largely in length and physicochemical properties. Their lengths ranged from 154 to 3576 amino acid residues. In terms of physicochemical properties (Additional file 1: Table S1), EXPASY analysis indicated that the TaWD40 protein sequences differed greatly in isoelectric point (pI, ranging from 4.23 to 10.99) and molecular weight (MW, 16.9–397.2 kDa).

### Chromosomal distribution and gene structure

**Fig. 1:**
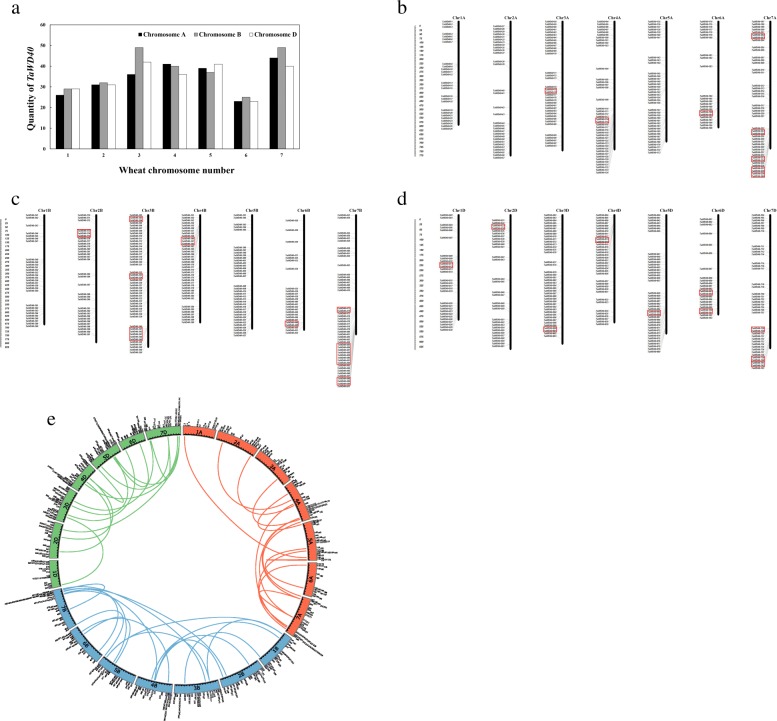
Genomic distributions of 743 *TaWD40*s on 21 wheat chromosomes. **(a)** Numbers of *TaWD40*s on each wheat chromosome. **(b)** “*TaWD40* distribution map” on 21 wheat chromosomes. Tandemly duplicated genes are marked by red boxes. The scale bar is shown in megabase (Mb). The picture was drawn by MapInspect. **(c)** Segmentally duplicated *TaWD40*s in wheat subgenomes A, B, and D. Arabic numerals represent the gene numbers of *TaWD40*s, and the different color lines indicate the synteny of *WD40*. The picture was drawn with Circos

The position of each *TaWD40* was determined by mapping its sequence to the corresponding chromosome of wheat cv. Chinese Spring (IWGSC RefSeq v1.0) via the BLAST program. The 743 *TaWD40*s were assigned to 21 wheat chromosomes. The positions of *TaWD40*s (Fig. 1) represented the relative locations in the genome instead of their real positions, considering that the length of each gap was directly set to 100 bp during sequence assembly. *TaWD40*s were extensively and unevenly distributed on the chromosomes. Subgenomes A, B, and D had 240, 261, and 242 *TaWD40*s, respectively. Each of chromosomes 3B and 7B had 49 genes, which had the largest number of *TaWD40*s. By contrast, chromosomes 6A and 6D had the least number of *TaWD40*s, and each had 23 genes (Fig. 1a). The distributions of *TaWD40*s showed that some genes accumulated on particular chromosomes. Numerous *TaWD40*s were distributed at the bottom of chromosomes, especially in 4A, 7A, 7B, 5D, and 7D. Relatively high densities were also detected at the top of chromosomes 4B and 4D. While relatively low densities of *TaWD40*s were mostly found at the top of chromosomes 6B, 1D, and 6D. Previous studies also demonstrated the widespread and uneven distribution features of *WD40*s on chromosomes in animals and plants [25, 26, 28, 29, 31].

To gain insights into the structures of *TaWD40*s, we analyzed their exon and intron organizations. The number of exons and introns in *TaWD40*s widely varied. The maximum number of exons contained in *TaWD40*s was 39 (38 introns), but only 1 exon and no intron existed in some genes. Among the *TaWD40*s, the genes containing 1 and 9 exons had the maximum number of members (59 genes), and 7–14 exons were present in about half of *TaWD40*s (Additional file 2: Figure S1, Additional file 1: Table S1).

### Gene duplication and genome synteny

Tandem and segmental duplication are key factors in gene family evolution to generate new gene members. In comparison with genomes in other grasses, inter- and intra-chromosomal duplications in wheat are more commonly detected through interspecific whole-genome analysis [33]. Thus, we investigated the segmental and tandem duplication events in the *WD40* gene family in wheat. In our study, 39 pairs of genes among 743 *TaWD40*s were identified as tandem duplications (Fig. 1b, Additional file 1: Table S2), and 46 pairs of genes might be related to segmental duplication events (Fig. 1c, Additional file 1: Table S3). Roughly one-to-one correspondences of these tandem duplication and segmental duplication events were observed in wheat A, B, and D subgenomes, that is, tandem duplication or segmental duplication events often occurred at the same locations in the three subgenomes of wheat.

The substitution rate of nonsynonymous (Ka) and synonymous (Ks) is the basis for evaluating the positive selection pressure of duplication events, where Ka/Ks = 1 denoted neutral selection, Ka/Ks < 1 indicated purifying selection, and Ka/Ks > 1 referred to positive selection. KaKs Calculator 2.0 was used to calculate Ka/Ks of duplicated *TaWD40*s. Ka/Ks of tandem duplications ranged from 0.03 to 1.22, and the mean value was 0.43, but the ratio of *TaWD40–273*/*TaWD40–274* was greater than 1 (Additional file 1: Table S2). Ka/Ks of 46 pairs of segmental duplication genes varied from 0.028 to 1.66, and the average was 0.39 (Additional file 1: Table S3). *TaWD40–463*/*TaWD40–492*, *TaWD40–364*/*TaWD40–443*, and *TaWD40–547*/*TaWD40–628* had Ka/Ks > 1, that is, 1.10571, 1.32411, and 1.66471, respectively. Therefore, duplication events played a pivotal role in the evolution of *TaWD40*s.

**Fig. 2:**
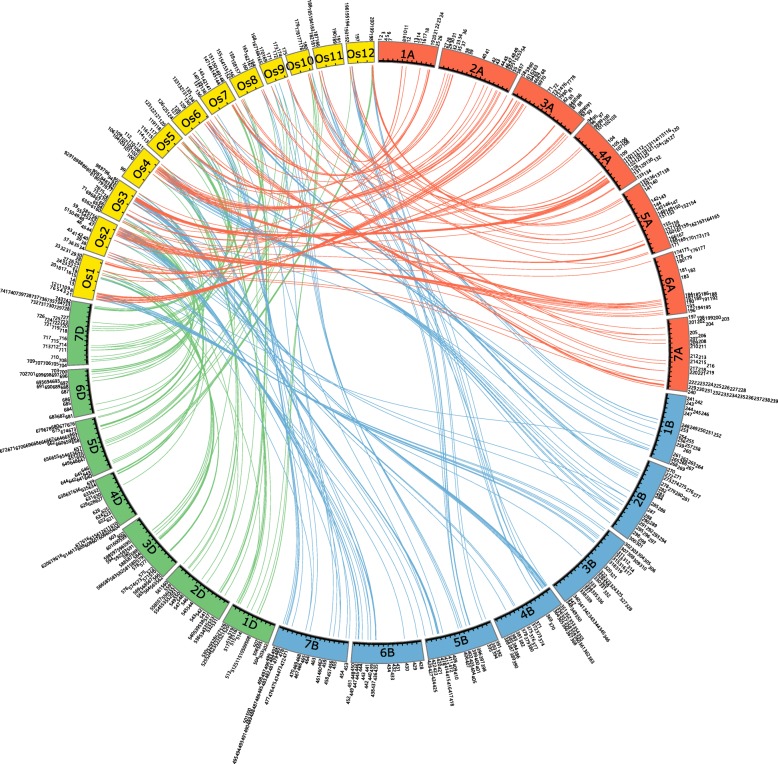
Syntenic relationships of WD40s between wheat (Ta) and rice (Os). The positions of all of the *WD40*s are depicted in the three subgenomes of wheat (red bands of A subgenome, blue bands of B subgenome, and green bands of D subgenome), rice (yellow bands). The Arabic numerals represent the gene numbers of *WD40* in wheat and rice. The different color lines indicate the synteny of *WD40* among wheat and rice. The picture was drawn with Circos

The syntenic relationships of *WD40*s between wheat and rice were analyzed to further study the evolution of *WD40*s. A total of 123 *OsWD40*s were identified to have synteny with *TaWD40*s (Fig. 2, Additional file 1: Table S4). The sequence similarities between the identified TaWD40s, OsWD40s, and AtWD40s were preliminarily examined by BLATP to identify the orthologous genes of *TaWD40*s in rice and *Arabidopsis* (Additional file 1: Table S5). For example, *TaWD40–123*, *TaWD40–352*, and *TaWD40–605* are identified as *TaGB1* and orthologous to *OsWD40–80* and *At4G34460.1*. *TaWD40–188* and *TaWD40–439* are orthologous to *OsWD40–50*, which are identified as *TaTTG1* [46].

### Classification and phylogenetic analysis of wheat WD40 proteins

**Fig. 3:**
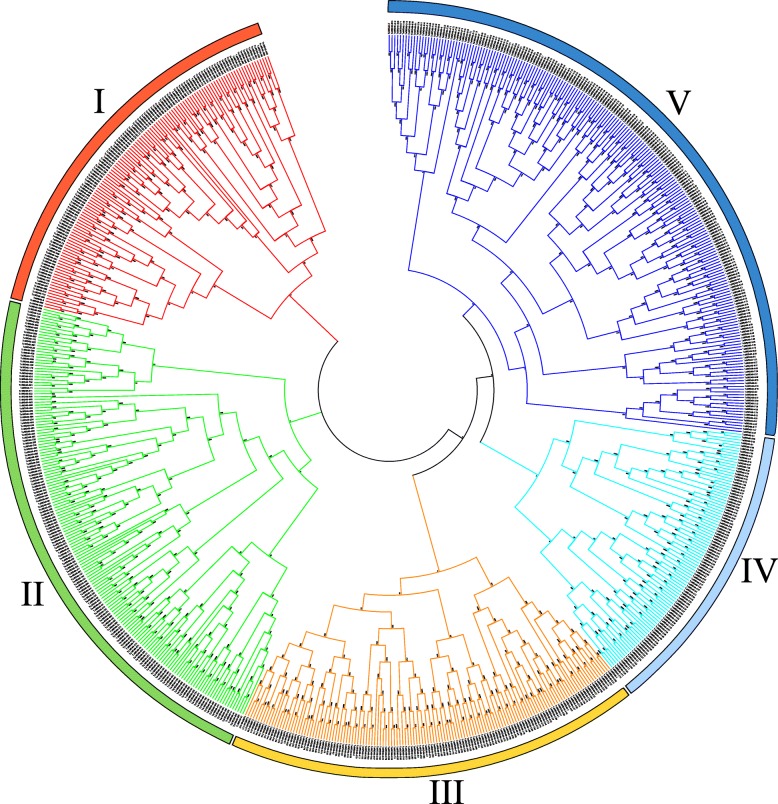
Phylogenetic classification of TaWD40 proteins. The phylogenetic tree was drawn with MEGA7 by using the neighbor-joining method with 1000 bootstrap replicates. All 743 TaWD40s were grouped into 5 clusters (Cluster I-V)

The protein sequences of the 743 identified TaWD40s were used to construct a phylogenetic tree via the neighbor-joining (N-J) method to analyze the evolutionary relationships of the WD40 family in wheat. In Fig. 3, the TaWD40s were divided into 5 major distinct clusters (Clusters I–V) containing 124, 173, 137, 89, and 220 proteins, respectively. The 743 TaWD40s were grouped into 11 subfamilies based on their domain compositions (Additional file 1: Table S1). The 478 TaWD40s containing only WD40 domains were classified as subfamily A. The 265 remaining TaWD40s comprising other additional domains were grouped into subfamilies B–K (Table 1).

**Table 1:** Domain composition and number of members of 11 subfamilies of 743 TaWD40s

Subfamily name	Domain composition	Number of members
Subfamily A	Only WD40 domain	478
Subfamily B	WD40 domain and LisH domain	42
Subfamily C	WD40 domain and UTP12, UTP13, UTP15 or UTP21	24
Subfamily D	WD40 domain and Coatomer WD/Coatomer (COPI) alpha subunit C-terminus	15
Subfamily E	WD40 domain and subunit C of CAF1 complex domains	13
Subfamily F	WD40 domain and NLE (NUC135) domain N terminal	6
Subfamily G	WD40 domain and protein kinase domain	60
Subfamily H	WD40 domain and Beige/BEACH domain	18
Subfamily I	WD40 domain and zinc finger domain	17
Subfamily J	WD40 domain and breast carcinoma amplified sequence 3 (BCAS3)	6
Subfamily K	WD40 domain and F-BOX, U-BOX or domains with unknown function	64

### Spatial and temporal expression profiles of *TaWD40*s

WD40 proteins have complicated structures and diverse functions, and studying their functions is difficult because of the allopolyploid characters of bread wheat. Their gene expression profile could provide useful information to reveal gene functions. The public RNA-seq data obtained from the expVIP website were used to analyze the spatial and temporal expression profiles of *TaWD40*s in wheat. The expression profiles covered 15 tissues during the entire life cycle of wheat (cv. Chinese Spring).

**Fig. 4:**
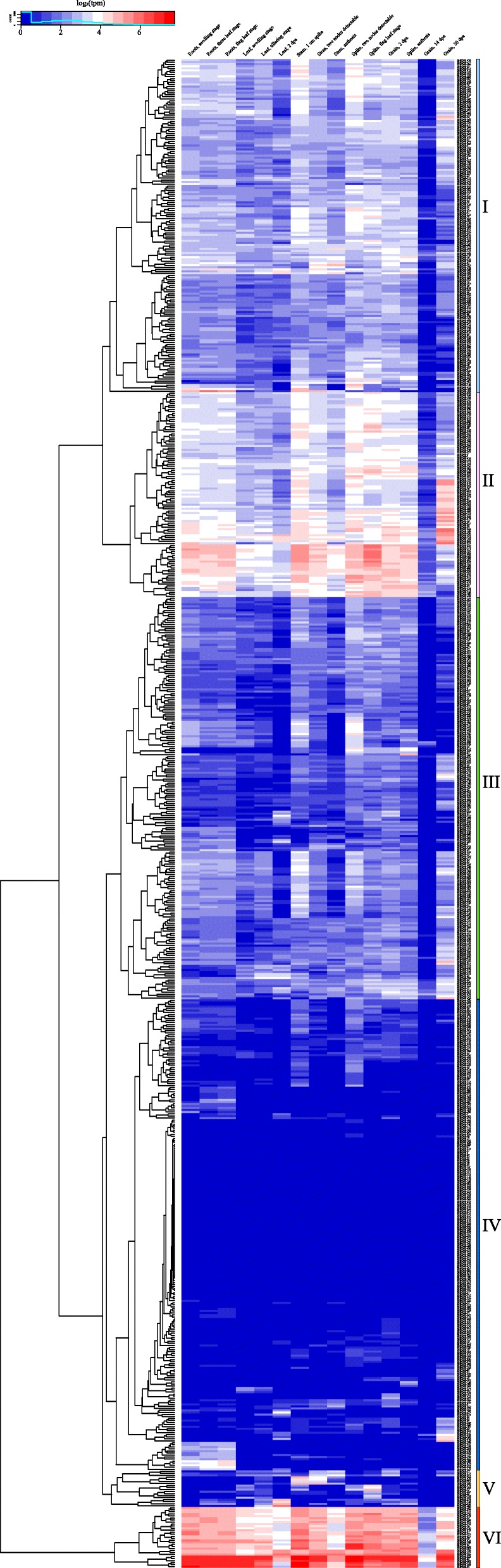
Spatial and temporal expression profiles of *TaWD40*. The heatmap was generated on the basis of the RNA-seq data and drawn with the R program. The color scale is shown at the upper left of the figure represents Log2 of transcript per million (TPM). Higher expression levels are shown in red, and lower expression levels are denoted in blue

The hierarchical cluster figure was generated by using the log2 of transcript per million (TPM) values of the 743 *TaWD40*s. The tissue expression profiles of *TaWD40*s were clustered into 6 groups (Groups I–VI) based on their expression characteristics (Fig. 4, Additional file 1: Table S6). Group I consisted of 164 genes, and the average expression levels in TPM ranged from 3.62 to 14.29 (average value 6.51). Group II comprised 101 genes with relatively high expression levels, and their average expression levels varied from 8.67 to 25.19 (average value 13.44). Group III was composed of 198 genes with low expression levels, and their average expression levels ranged from 1.61 to 6.29 (average value 3.58). Group IV included 232 genes that were barely expressed in almost all of the tested tissues, and their average value was 0.79. Group V had 18 genes with relatively low expression levels in most tissues except in some individual tissues. Group VI contained the 30 remaining genes with high expression levels in all of the 15 tissues, and their expression levels ranged from 24.80 to 121.89 with an average value of 44.73.

The expression profiles of *TaWD40*s are similar to *OsWD40*s [31], and multiple expression characteristics demonstrate the diverse functions of some TaWD40s in wheat growth and development. For example, *TaWD40–289* belonging to Group I was remarkably and highly expressed in roots and stems but was relatively weakly expressed in other tissues, implying that this gene might play an important role in root and stem development. Group IV members, such as *TaWD40–21*, *TaWD40–263*, and *TaWD40–522* (homologous genes on subgenomes A, B, and D), almost had no expression in all of the 15 analyzed tissues, and they might participate in some special physiological and biological processes under certain conditions. Group VI genes, such as *TaWD40–82*, *TaWD40–332*, and *TaWD40–589* (homologous genes on subgenomes A, B, and D), which were identified as guanine nucleotide-binding protein beta subunit-like genes, might perform housekeeping functions and had the highest average expression levels in all of the tissues among 743 analyzed genes.

### Expression profiles of *TaWD40*s under biotic and abiotic stresses

The RNA-seq data were downloaded from the expVIP website based on IWGSC 1.0 annotation. The differential expression of *TaWD40*s under biotic stresses (powdery mildew pathogen and stripe rust pathogen infection) and abiotic stresses (cold, heat and drought) was analyzed, and their MA plots were drawn (Additional file 2: Figure S2, Additional file 1: Table S7). The upregulated and downregulated *TaWD40*s were also counted under different stresses (Table 2). Our results revealed 92 significantly upregulated *TaWD40*s and 73 significantly downregulated *TaWD40*s in wheat shoots at the three-leaf stage under cold stress. Additionally, the number of differentially expressed *TaWD40*s under heat stress was more than that under drought stress. More *TaWD40*s were downregulated compared with the upregulated ones. The number of stress-responsive *TaWD40*s under 6 h of treatment was more than that under 1 h of treatment. Furthermore, the responses of TaWD40s to biotic stresses mainly occurred at the early stage of pathogen infection, and the number of differentially expressed genes gradually decreased as time progressed. The number of the upregulated *TaWD40*s exposed to powdery mildew pathogen E09 was higher than that of the upregulated *TaWD40*s infected by stripe rust pathogen CYR31.

**Table 2:** Number of stress-responsive *TaWD40*s under different treatment conditions

Stress, treatment time	Up^a^, percentage	Down^a^, percentage	Low counts, percentage	Nonzero total read count	Study title
Cold, 2 week	92, 14%	73, 11%	51, 7.8%	657	SRP043554
Heat stress, 1 h	97, 15%	78, 12%	24, 3.7%	647	SRP045409
Heat stress, 6 h	188, 29%	160, 25%	13, 2%	651	SRP045409
Drought stress, 1 h	69, 11%	21, 3.3%	62, 9.6%	646	SRP045409
Drought stress, 6 h	103, 16%	100, 15%	11, 1.7%	646	SRP045409
Drought and heat stresses, 1 h	143, 22%	115, 18%	37, 5.8%	641	SRP045409
Drought and heat stresses, 6 h	181, 28%	150, 23%	0, 0%	646	SRP045409
Powdery mildew pathogen E09, 24 h	25, 3.9%	13, 2%	243, 38%	635	SRP041017
Powdery mildew pathogen E09, 48 h	6, 0.9%	16, 2.5%	0, 0%	636	SRP041017
Powdery mildew pathogen E09, 72 h	2, 0.3%	11, 1.7%	87, 14%	644	SRP041017
Stripe rust pathogen CYR31, 24 h	12, 1.9%	15, 2.4%	97, 15%	632	SRP041017
Stripe rust pathogen CYR31, 48 h	1, 0.2%	3, 0.5%	0, 0%	632	SRP041017
Stripe rust pathogen CYR31, 72 h	3, 0.5%	4, 0.6%	0, 0%	630	SRP041017

^a^Upregulated and downregulated *TaWD40*s were counted when the padj (adjusted *p* value) was less than 0.05

### Expression profiles of *TaWD40*s during seed development

Some *WD40*s are essential for seed development; for instance, TTG1 participates in the pigmentation of testa and the development of trichomes in *A. thaliana* [47, 48] and sorghum [49]. The tissue expression of *TaWD40*s indicates that many genes are specifically expressed in spikes and grains, suggesting their important roles in wheat seed development. Hence, 26 *TaWD40*s were randomly selected from the 5 distinct clusters of the phylogenetic tree to analyze their expression characteristics during wheat seed development through qRT-PCR.

**Fig. 5:**
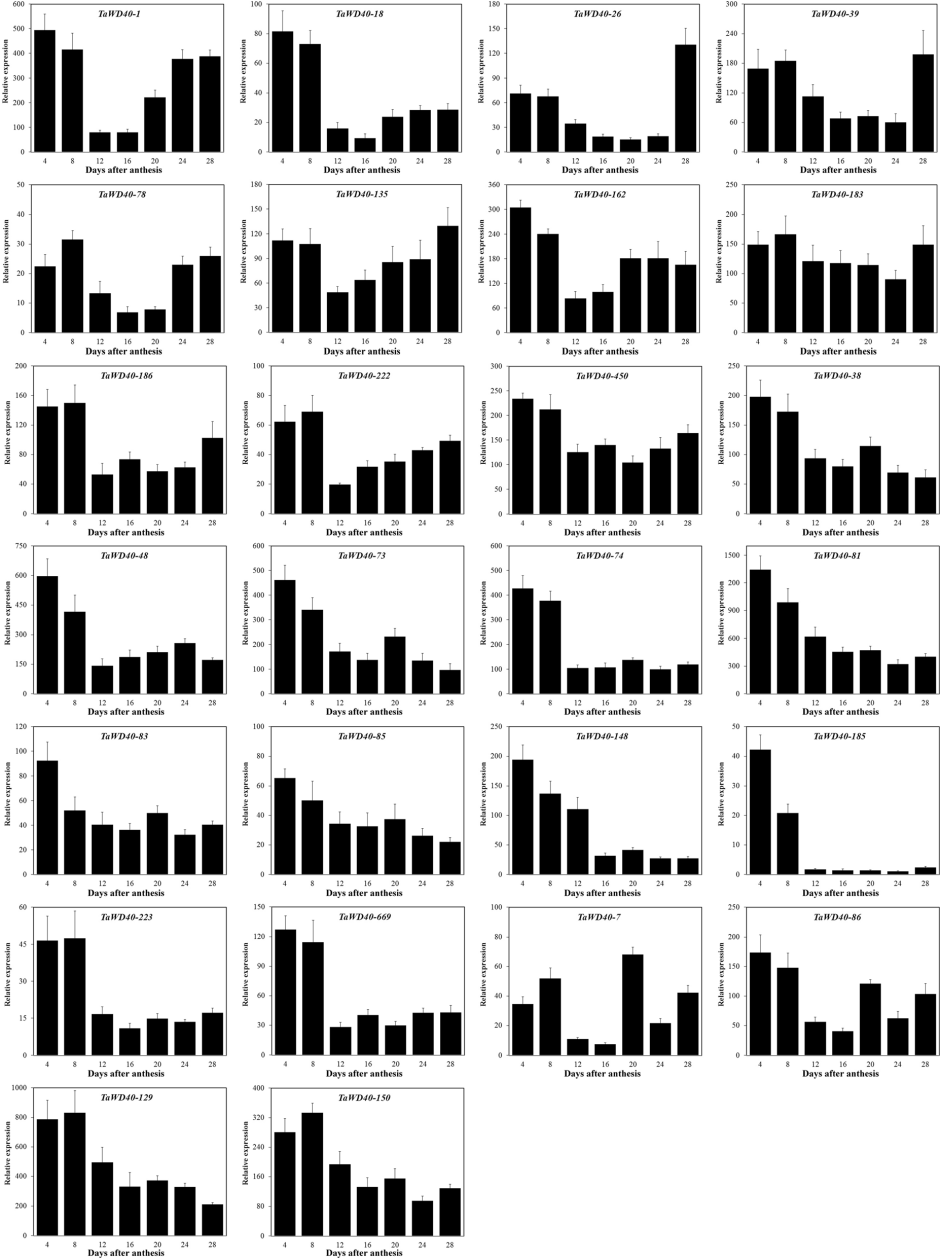
Expression profiles of 26 selected *TaWD40*s in the seed development of *T. aestivum* L. cv. Chinese Spring. Y-axis, relative expression levels; X-axis, days after anthesis. The error bars represent standard deviation (S.D.) calculated from three replications. The gene expression levels are normalized to the internal control of *TaActin*

We sampled the developing seeds every 4 days after anthesis for a total of 7 sampling times. Among the 26 selected *TaWD40*s (Fig. 5, Additional file 2: Figure S3, Additional file 1: Table S8), the expression levels of *TaWD40–129* and *TaWD40–150* increased rapidly during the early stages of seed development and gradually decreased with seed maturity. Furthermore, 11 *TaWD40*s (*TaWD40–1*, *TaWD40–18*, *TaWD40–26*, *TaWD40–39*, *TaWD40–78*, *TaWD40–135*, *TaWD40–162*, *TaWD40–183*, *TaWD40–186*, *TaWD40–222*, and *TaWD40–450*) shared the same expression pattern; that is, their expression levels were downregulated at the early stages of seed development, but their expression levels increased gradually 12 days after anthesis. The expression levels of *TaWD40–38*, *TaWD40–48*, *TaWD40–73*, *TaWD40–74*, *TaWD40–81*, *TaWD40–83*, *TaWD40–85*, *TaWD40–148*, *TaWD40–185*, *TaWD40–223*, and *TaWD40–669* continually decreased from relatively high expression levels (4 days after anthesis) during wheat seed development. The expression levels of *TaWD40–7* and *TaWD40–86* fluctuated and had no obvious expression trends.

## Discussion

### Expansions of *WD40*s in wheat

*WD40* family expansion is a result of tandem and segmental duplications. In our study, tandem duplications led to 5.2% (39 of 743) of newly produced genes, and segmental duplication events contributed to 6.2% (46 of 743) of the newly generated genes. Different subfamilies had various effects on wheat gene expansion events. In particular, tandem duplications mainly originated from subfamily A (41%, 16 of 39) and subfamily G (46%, 18 of 39) grouped in Cluster II. Segmental duplication gene pairs were mainly from subfamily A (65%, 30 of 46). The homologous genes of *TaWD40–103* and *TaWD40–135* located on subgenome A were *TaWD40–627* and *TaWD40–640* on subgenome D, and these two pairs of genes were identified as segmental duplication genes belonging to subfamilies A and B, respectively. The N-terminals of TaWD40–103 and TaWD40–627 had similar multiple mutations, and the LisH domain could not be formed. As such, they were grouped into subfamily A. Thus, duplication and homologous recombination were the major primary driving forces of the evolution of the *TaWD40* family. Allopolyploidy and high-level gene duplication (inter and intra-chromosomal duplications) provided a large genome with numerous functional genes for the adaptation of wheat in complicated various environments.

### Expression profile analysis of *TaWD40*s

Tissue expression profile analysis revealed that many *TaWD40*s were differentially expressed in spike and seed development (Fig. 4), indicating that TaWD40s might participate in seed development. TaWD40–161 was identified as the orthologous protein of *Arabidopsis* JINGUBANG, which is a negative regulator of pollen germination [50]. *TaWD40–161* was specifically and highly expressed in spikes during anthesis but was almost not expressed in other tissues. TaWD40–161 might play a pivotal role in anther development. The qRT-PCR results verified the special expression profiles of some *TaWD40*s during seed development (Fig. 5). The RNA-seq data of tissue expression profile analysis exhibited the expression levels of *TaWD40*s in wheat seed grown for 2, 14, and 30 days after anthesis. Interestingly, the expression levels of almost all of *TaWD40*s in the wheat seed 14 days after anthesis were lower than those in the wheat seed 2 and 30 days after anthesis, and this expression characteristic was confirmed by the qRT-PCR results. The expression levels of most selected *TaWD40*s gradually decreased at the early stage of wheat seed development. The expression levels of *TaWD40–135*, *TaWD40–26*, and *TaWD40–162* decreased and then significantly increased during wheat seed development. TaWD40–135 (containing the LisH domain at N-terminus and the bromodomain at C-terminus), TaWD40–26 (containing the NLE domain), and TaWD40–162 (containing BEACH domains) might participate in wheat seed development. TaWD40–1 and TaWD40–78 were identified as RAE1-like protein, which is an essential mitotic checkpoint regulator [51].

*WD40*s participate in responses to various abiotic stresses [52–55]. For instance, *TaWD40D* (identified as *TaWD40–614* in this work), which is homologous to *TaWD40–114* and *TaWD40–362* (Additional file 1: Table S9-S11), functions as a positive regulator of plant responses to salt and osmotic stresses [53]. In our study, the upregulated expression of *TaWD40–362* under cold, heat, and drought stresses validated that it was implicated in multiple abiotic stress responses. RNA-seq data analysis revealed that a number of *TaWD40*s responded to multiple abiotic stresses, and the number of the upregulated genes was larger than that of the downregulated ones. The number of responsive *TaWD40*s remarkably increased as the treatment time was extended. Conversely, only a few *TaWD40*s significantly responded to biotic stresses at the early stage of treatments.

## Conclusions

In this study, 743 *TaWD40*s distributed on subgenomes A, B, and D were identified and analyzed on a genome-wide scale. All of the genes were distributed extensively and unevenly on every chromosome of each subgenome, but they had one-to-one homology relationship at a subgenome level. Phylogenetic analysis and conserved domain prediction revealed that 743 TaWD40s were arranged in 5 main distinct clusters and grouped into 11 subfamilies. Synteny analysis indicated that *WD40*s in wheat were highly homologous to those in rice. Sequence analysis suggested that segmental duplication and tandem duplication were the major driving forces of *TaWD40* family evolution. Homologous recombination was also essential for gene evolution.

The expression profiles of *TaWD40*s were analyzed by using RNA-seq data, and the results indicated that most *TaWD40*s were expressed in the entire life cycle of wheat. Some tissue-specific expression genes might participate in the development of spikes and seeds. The qRT-PCR analysis confirmed the crucial roles of some TaWD40s in wheat seed development. The expression profiles of *TaWD40*s under different stresses indicated that a large number of TaWD40s were involved in responses to stresses of cold, heat, drought, and powdery mildew infection in wheat. Our study provided a reference for the further functional investigation of these selected candidate TaWD40 proteins.

## Methods

### Identification of TaWD40 proteins

The HMM profile of the WD40 domain (PF00400) was downloaded from Pfam (http://pfam.xfam.org/family/PF00400) to identify all of the WD40 proteins in wheat, and the whole genome sequence of wheat cv. Chinese Spring (IWGSC RefSeqv1.0) was downloaded from URGI (https://wheat-urgi.versailles.inra.fr/Seq-Repository/Assemblies). All of the WD40 proteins were identified by using the default settings of hmmsearch program of the HMMER3.0 software (http://hmmer.org/download.html), and the redundant sequences among them were deleted by utilizing the perl program. The SMART website (http://smart.embl-heidelberg.de) [44] and the HMMER website (https://www.ebi.ac.uk/Tools/hmmer/) [43] were used to confirm all the TaWD40s containing WD40 repeat. In addition to the WD40 domain, other conserved motifs in these genes were identified.

### Structural analysis of *TaWD40*s

The coding sequence (CDS), protein sequence, and genomic sequence of the identified *TaWD40*s were obtained from IWGSC RefSeq v1.0 (https://wheat-urgi.versailles.inra.fr/Seq-Repository/Assemblies). An exon/intron map was drawn by uploading their CDS and genomic sequences to Gene Structure Display Server (GSDS 2.0, http://gsds.cbi.pku.edu.cn/) [56]. Theoretical pI and MW were analyzed (https://web.expasy.org/compute_pi/) [57, 58].

### Chromosomal distribution, gene duplication, and synteny analysis

The location of each *WD40* on the 21 wheat chromosomes was mapped to IWGSC RefSeq v1.0 (cv. Chinese_Spring) by using Blast programs (https://blast.ncbi.nlm.nih.gov/Blast.cgi) [59], and a physical map was drawn with MapInspect. The following criteria were used to identify the tandem duplication events of *TaWD40*s: 1) alignment length was over 80% of the full length of the gene, 2) aligned region had an identity over 80%, and 3) no genes were inserted between them. Segmental duplication was defined as follows: 1) alignment length was longer than 1 kb, and 2) aligned region had an identity of > 90% [60–63]. The segmental duplications in three subgenomes were separately identified, considering that wheat is a hexaploid. MCScanX was used to analyze the synteny of WD40s between wheat and rice [64, 65], and figures were drawn with Circos v0.69 [66]. Ka/Ks was calculated with KaKs Calculator 2.0 (https://sourceforge.net/projects/kakscalculator2/) [67].

### Phylogenetic analysis

The WD40 protein sequences of wheat were imported to Clustal X2 (http://www.clustal.org/clustal2/) [68], and complete alignment was conducted by using the default settings. The alignment results were imported to MEGA7 (https://www.megasoftware.net/) [69] to construct an unrooted phylogenetic tree by using N-J method with a bootstrap of 1000 replicates.

### Expression profile analysis

The RNA-seq data were downloaded from the expVIP website (http://www.wheat-expression.com/) to analyze the spatial and temporal expression profiles of *WD40*s in wheat [70, 71]. The study title was “choulet_URGI”. Two biological replicates with 15 tissue types of wheat (cv. Chinese Spring) were used. A heatmap was drawn by using R program (https://www.R-project.org/) gplots package. The RNA-seq data (study title “SRP043554,” “SRP045409,” and “SRP041017”) were downloaded from the expVIP website to examine the expression profiles of *TaWD40*s under different stresses (cold, heat and drought, powdery mildew pathogen, and stripe rust pathogen). R program DESeq2 package was utilized to evaluate the differential expression of *TaWD40*s under stresses and to draw MA plots.

For qRT-PCR analysis, the seeds of wheat cv. Chinese Spring were sampled every 4 days after anthesis for 7 continuous times. Target materials were quick frozen in liquid nitrogen and then transferred to a refrigerator at − 80 °C. Total RNA was collected with a plant tissue total RNA extraction kit (Zomanbio, Beijing, China), and the first cDNA chain was synthesized using a FastKing RT kit with gDNase (Tiangen, Beijing, China). The qRT-PCR analysis was performed using AceQ qPCR SYBR Green Master Mix (Vazyme, Nanjing, China) on a qRT-PCR machine (Bio-Rad, Hercules, CA, USA). The primer sequences used for the expression profile analysis are presented in Additional file 1: Table S8. The wheat housekeeping gene *TaActin* (accession No. AB181991.1) was used as a reference gene.

## Additional files

Additional file 1: **Table S1.** Characteristic features of 743 *TaWD40*s. **Table S2.** KaKs ratios of tandemly duplicated *TaWD40*s. **Table S3.** KaKs ratios of segmentally duplicated *TaWD40*s. **Table S4.** Syntenic relationships of *WD40*s between wheat and rice. **Table S5.** Homologous WD40s among wheat, rice, and *Arabidopsis*. **Table S6.** The expression levels of TaWD40s for spatial and temporal expression profiles analysis. **Table S7.** Significantly and differentially expressed *TaWD40*s under biotic and abiotic stresses. **Table S8.** Primers used for qRT-PCR. **Table S9.** Amino acid sequences of 743 TaWD40s. **Table S10**. Coding sequences of 743 *TaWD40*s. **Table S11**. Corresponding table for homologous *TaWD40*s located on 21 wheat chromosomes. **Table S12**. Functional annotation of *TaWD40*s from IWGSC RefSeqv1.0. (ZIP 1478 kb)

Additional file 2: **Figure S1.** Exon/intron organizations of 743 *TaWD40*s. Solid yellow boxes and black lines indicate exons and introns, respectively. The scale is shown at the bottom of the figure. **Figure S2.** MA plots of differentially expressed *TaWD40*s under biotic and abiotic stresses. MA plots were generated with DESeq2 version 1.20.0. Points are highlighted in red when padj is less than 0.05, representing significantly and differentially expressed *TaWD40*s. Points falling outside of 2 to − 2 log fold are plotted as open triangles pointing either up or down. **(a)** Cold stress for 2 weeks, **(b)** Heat stress for 1 h, **(c)** Heat stress for 6 h, **(d)** Drought stress for 1 h, **(e)** Drought stress for 6 h, **(f)** Drought and heat stresses for 1 h, **(g)** Drought and heat stresses for 6 h, **(h)** Infection of powdery mildew pathogen (E09) for 24 h, **(i)** Infection of powdery mildew pathogen (E09) for 48 h, **(j)** Infection of powdery E09 for 72 h, **(k)** Infection of stripe rust pathogen (CYR31) for 24 h, **(l)** Infection of CYR31 for 48 h, and **(m)** Infection of CYR31 for 72 h. **Figure S3**. Standard and dissociation curves of qRT-PCR. (ZIP 9762 kb)

## Abbreviations

BCAS3: Breast carcinoma amplified sequence 3
CAF1: CCR4-associated factor 1
CDS: coding sequence
DDB1: DNA damage binding protein 1
FAO: Food and Agriculture Organization
GEO: Gene expression omnibus
GSDS: Gene Structure Display Server
HMM: The hidden Markov model
IWGSC: The International Wheat Genome Sequencing Consortium
Ka: non-synonymous
Ks: synonymous
MW: Molecular weight
N-J: Neighbor-Joining
padj: adjusted *p* value
pI: Isoelectric point
TPM: transcript per million
