# Correction to: Genome-wide identification and analysis of WD40 proteins in wheat (*Triticum aestivum* L.)

**DOI:** 10.1186/s12864-018-5252-2

**Published:** 2018-11-29

**Authors:** Rui Hu, Jie Xiao, Ting Gu, Xiaofen Yu, Yang Zhang, Junli Chang, Guangxiao Yang, Guangyuan He

**Affiliations:** The Genetic Engineering International Cooperation Base of Chinese Ministry of Science and Technology, Key Laboratory of Molecular Biophysics of Chinese Ministry of Education, College of Life Science and Technology, Huazhong University of Science and Technology (HUST), Wuhan, 430074 China

## Correction

Following the publication of this article [1], the authors reported the following errors:

In Fig. 1a, “Chromosome A, Chromosome B, and Chromosome D” should be “Subgenome A, Subgenome B, and Subgenome D”.

**Fig. 1:**
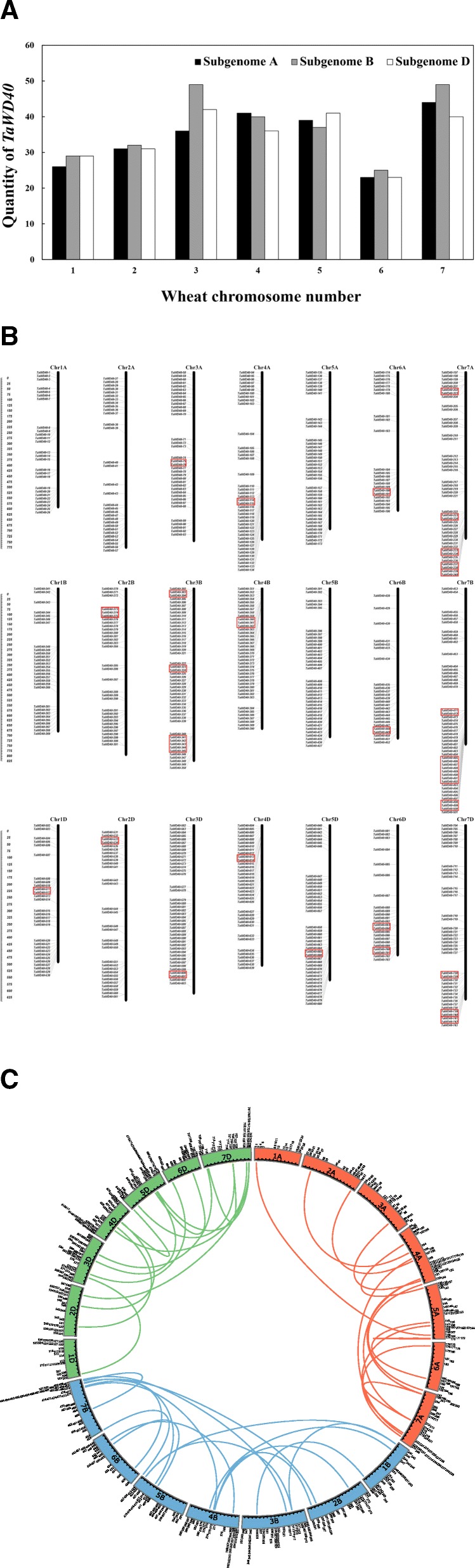
Genomic distributions of 743 *TaWD40*s on 21 wheat chromosomes. (**a**) Numbers of *TaWD40*s on each wheat chromosome. (**b**) “*TaWD40* distribution map” on 21 wheat chromosomes. Tandemly duplicated genes are marked by red boxes. The scale bar is shown in megabase (Mb). The picture was drawn by MapInspect. (**c**) Segmentally duplicated *TaWD40*s in wheat subgenomes A, B, and D. Arabic numerals represent the gene numbers of *TaWD40*s, and the different color lines indicate the synteny of *WD40*. The picture was drawn with Circos

In Fig. 1, Figures “1b”, “1c” and “1d” should be labeled together as Figure “1b”, and Figure “1e” should be labeled as “Fig. 1c”.

The authors regret any confusion caused by this error. The correct version of Fig. 1 is included in this Correction article.
