# Supplementary figures and images for: Genome-wide identification and analysis of WD40 proteins in wheat (Triticum aestivum L.)
Source: BMC Genomics. 2018 Nov 6;19:803. doi: 10.1186/s12864-018-5157-0 (PMC6219084; doi:10.1186/s12864-018-5157-0)

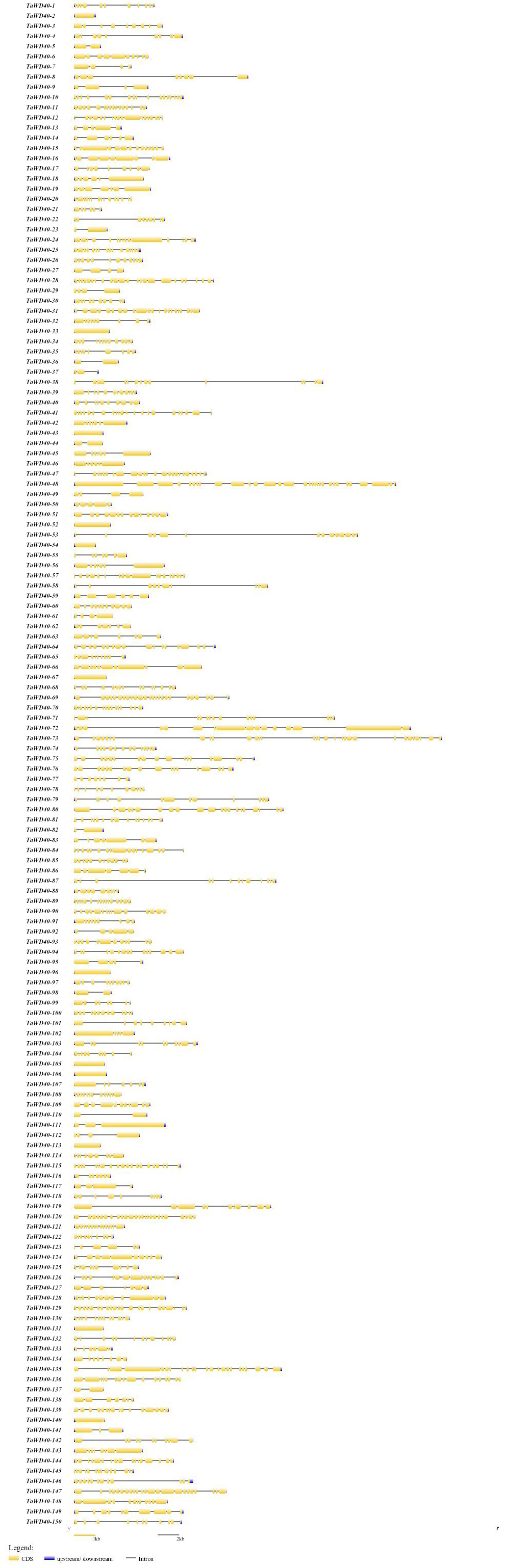

Supplement: Supplementary file 2 — Figure S1. Exon/intron organizations of 743 TaWD40s. Solid yellow boxes and black lines indicate exons and introns, respectively. The scale is shown at the bottom of the figure. Figure S2. MA plots of differentially expressed TaWD40s under biotic and abiotic stresses. MA plots were generated with DESeq2 version 1.20.0. Points are highlighted in red when padj is less than 0.05, representing significantly and differentially expressed TaWD40s. Points falling outside of 2 to − 2 log fold are plotted as open triangles pointing either up or down. (a) Cold stress for 2 weeks, (b) Heat stress for 1 h, (c) Heat stress for 6 h, (d) Drought stress for 1 h, (e) Drought stress for 6 h, (f) Drought and heat stresses for 1 h, (g) Drought and heat stresses for 6 h, (h) Infection of powdery mildew pathogen (E09) for 24 h, (i) Infection of powdery mildew pathogen (E09) for 48 h, (j) Infection of powdery E09 for 72 h, (k) Infection of stripe rust pathogen (CYR31) for 24 h, (l) Infection of CYR31 for 48 h, and (m) Infection of CYR31 for 72 h. Figure S3. Standard and dissociation curves of qRT-PCR. (ZIP 9762 kb) [file 12864_2018_5157_MOESM2_ESM.zip › Figure S1-1.jpg]

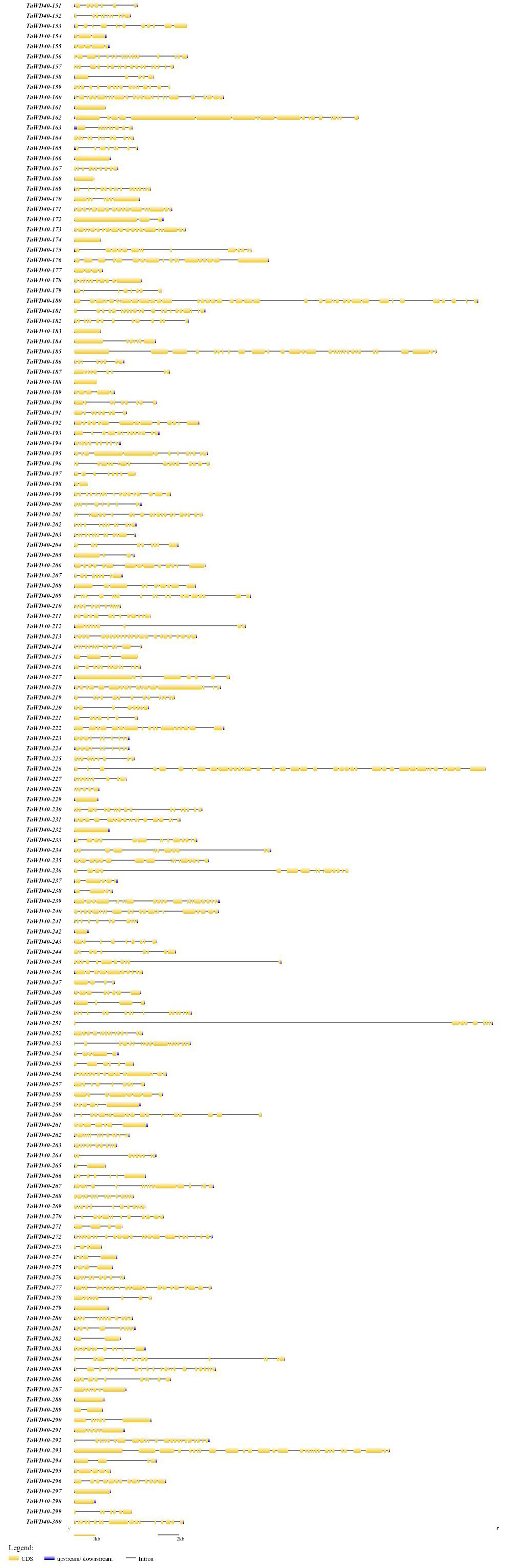

Supplement: Supplementary file 2 — Figure S1. Exon/intron organizations of 743 TaWD40s. Solid yellow boxes and black lines indicate exons and introns, respectively. The scale is shown at the bottom of the figure. Figure S2. MA plots of differentially expressed TaWD40s under biotic and abiotic stresses. MA plots were generated with DESeq2 version 1.20.0. Points are highlighted in red when padj is less than 0.05, representing significantly and differentially expressed TaWD40s. Points falling outside of 2 to − 2 log fold are plotted as open triangles pointing either up or down. (a) Cold stress for 2 weeks, (b) Heat stress for 1 h, (c) Heat stress for 6 h, (d) Drought stress for 1 h, (e) Drought stress for 6 h, (f) Drought and heat stresses for 1 h, (g) Drought and heat stresses for 6 h, (h) Infection of powdery mildew pathogen (E09) for 24 h, (i) Infection of powdery mildew pathogen (E09) for 48 h, (j) Infection of powdery E09 for 72 h, (k) Infection of stripe rust pathogen (CYR31) for 24 h, (l) Infection of CYR31 for 48 h, and (m) Infection of CYR31 for 72 h. Figure S3. Standard and dissociation curves of qRT-PCR. (ZIP 9762 kb) [file 12864_2018_5157_MOESM2_ESM.zip › Figure S1-2.jpg]

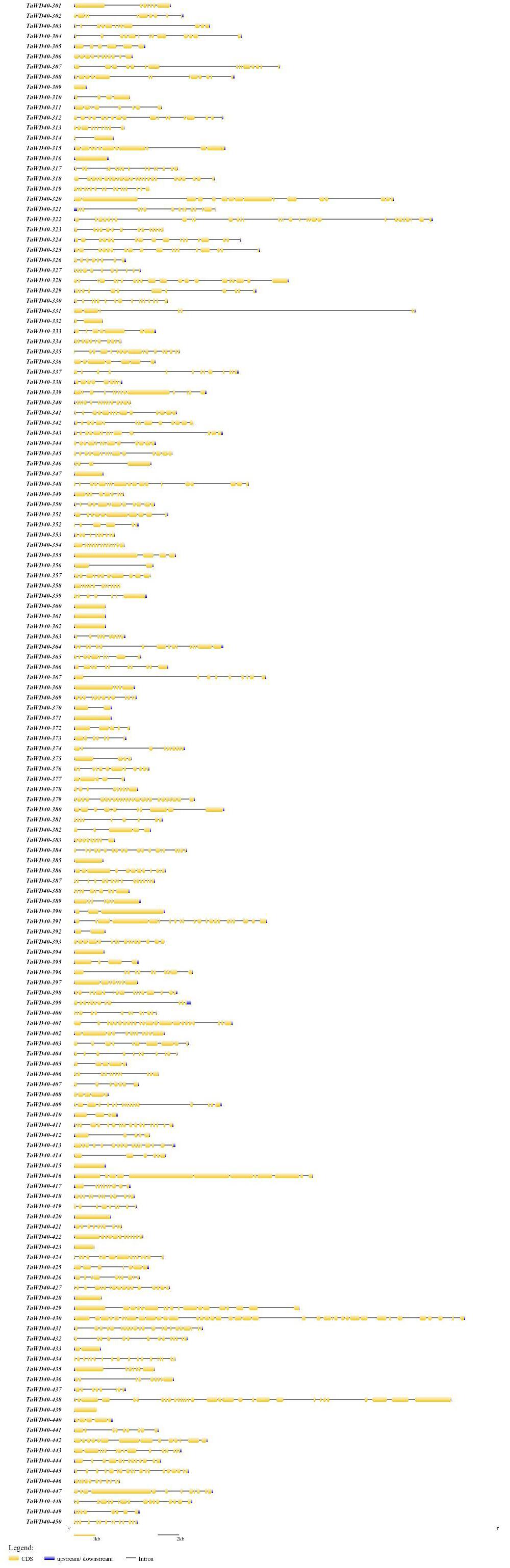

Supplement: Supplementary file 2 — Figure S1. Exon/intron organizations of 743 TaWD40s. Solid yellow boxes and black lines indicate exons and introns, respectively. The scale is shown at the bottom of the figure. Figure S2. MA plots of differentially expressed TaWD40s under biotic and abiotic stresses. MA plots were generated with DESeq2 version 1.20.0. Points are highlighted in red when padj is less than 0.05, representing significantly and differentially expressed TaWD40s. Points falling outside of 2 to − 2 log fold are plotted as open triangles pointing either up or down. (a) Cold stress for 2 weeks, (b) Heat stress for 1 h, (c) Heat stress for 6 h, (d) Drought stress for 1 h, (e) Drought stress for 6 h, (f) Drought and heat stresses for 1 h, (g) Drought and heat stresses for 6 h, (h) Infection of powdery mildew pathogen (E09) for 24 h, (i) Infection of powdery mildew pathogen (E09) for 48 h, (j) Infection of powdery E09 for 72 h, (k) Infection of stripe rust pathogen (CYR31) for 24 h, (l) Infection of CYR31 for 48 h, and (m) Infection of CYR31 for 72 h. Figure S3. Standard and dissociation curves of qRT-PCR. (ZIP 9762 kb) [file 12864_2018_5157_MOESM2_ESM.zip › Figure S1-3.jpg]

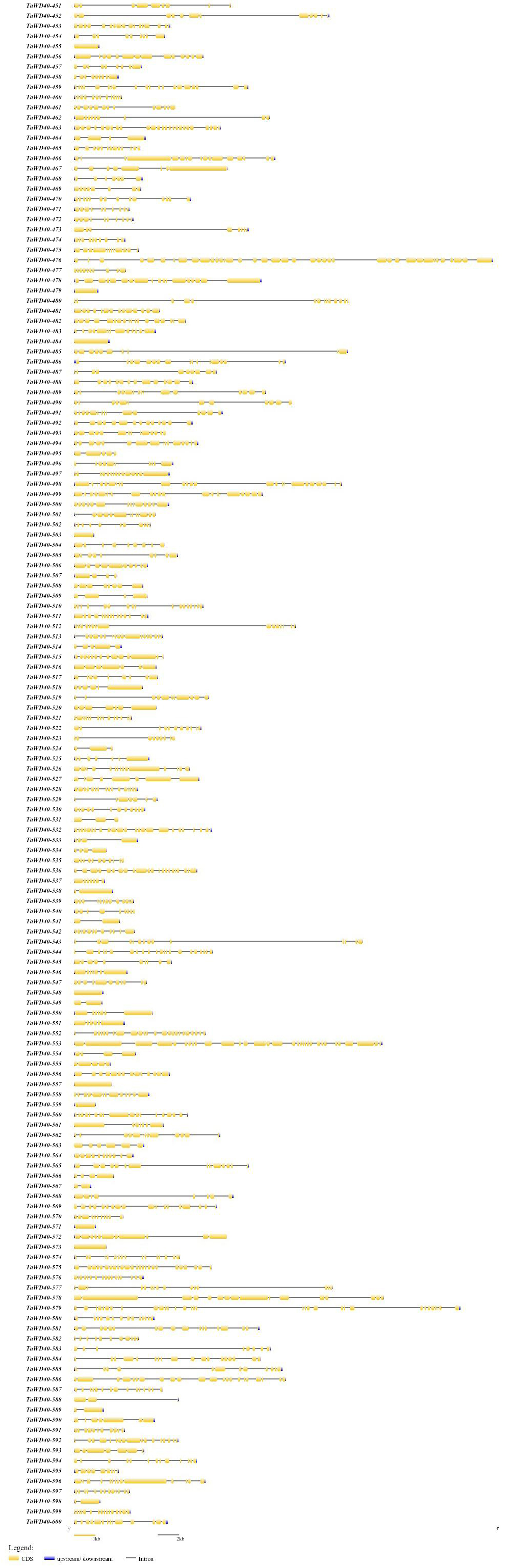

Supplement: Supplementary file 2 — Figure S1. Exon/intron organizations of 743 TaWD40s. Solid yellow boxes and black lines indicate exons and introns, respectively. The scale is shown at the bottom of the figure. Figure S2. MA plots of differentially expressed TaWD40s under biotic and abiotic stresses. MA plots were generated with DESeq2 version 1.20.0. Points are highlighted in red when padj is less than 0.05, representing significantly and differentially expressed TaWD40s. Points falling outside of 2 to − 2 log fold are plotted as open triangles pointing either up or down. (a) Cold stress for 2 weeks, (b) Heat stress for 1 h, (c) Heat stress for 6 h, (d) Drought stress for 1 h, (e) Drought stress for 6 h, (f) Drought and heat stresses for 1 h, (g) Drought and heat stresses for 6 h, (h) Infection of powdery mildew pathogen (E09) for 24 h, (i) Infection of powdery mildew pathogen (E09) for 48 h, (j) Infection of powdery E09 for 72 h, (k) Infection of stripe rust pathogen (CYR31) for 24 h, (l) Infection of CYR31 for 48 h, and (m) Infection of CYR31 for 72 h. Figure S3. Standard and dissociation curves of qRT-PCR. (ZIP 9762 kb) [file 12864_2018_5157_MOESM2_ESM.zip › Figure S1-4.jpg]

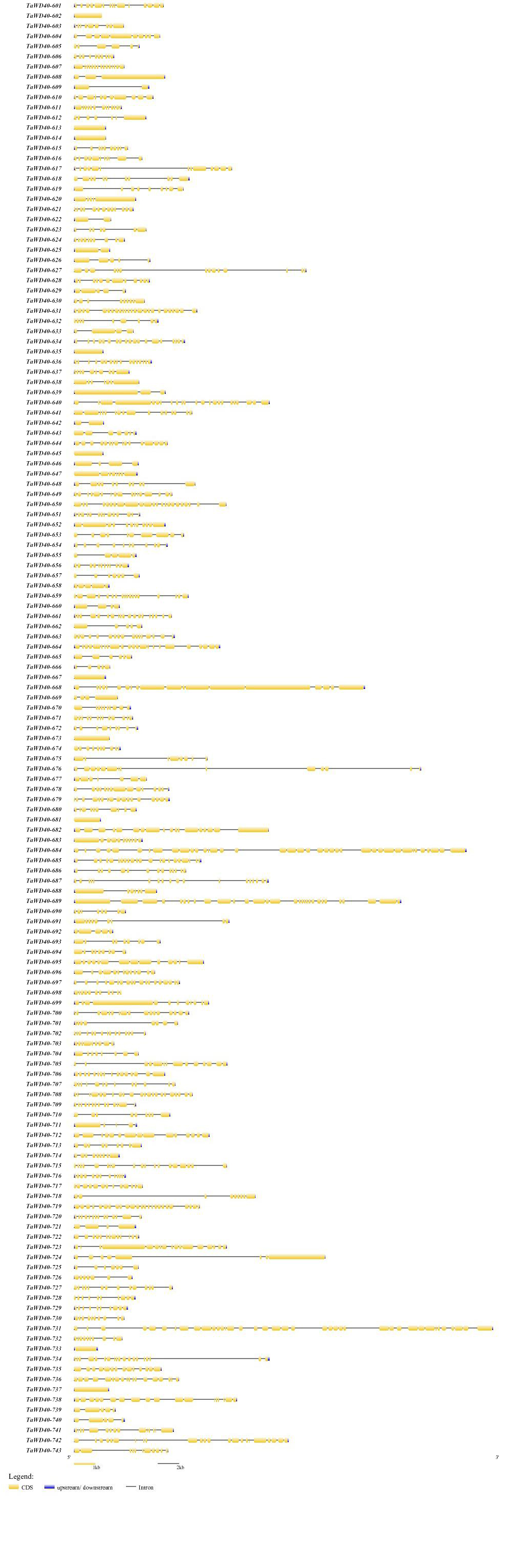

Supplement: Supplementary file 2 — Figure S1. Exon/intron organizations of 743 TaWD40s. Solid yellow boxes and black lines indicate exons and introns, respectively. The scale is shown at the bottom of the figure. Figure S2. MA plots of differentially expressed TaWD40s under biotic and abiotic stresses. MA plots were generated with DESeq2 version 1.20.0. Points are highlighted in red when padj is less than 0.05, representing significantly and differentially expressed TaWD40s. Points falling outside of 2 to − 2 log fold are plotted as open triangles pointing either up or down. (a) Cold stress for 2 weeks, (b) Heat stress for 1 h, (c) Heat stress for 6 h, (d) Drought stress for 1 h, (e) Drought stress for 6 h, (f) Drought and heat stresses for 1 h, (g) Drought and heat stresses for 6 h, (h) Infection of powdery mildew pathogen (E09) for 24 h, (i) Infection of powdery mildew pathogen (E09) for 48 h, (j) Infection of powdery E09 for 72 h, (k) Infection of stripe rust pathogen (CYR31) for 24 h, (l) Infection of CYR31 for 48 h, and (m) Infection of CYR31 for 72 h. Figure S3. Standard and dissociation curves of qRT-PCR. (ZIP 9762 kb) [file 12864_2018_5157_MOESM2_ESM.zip › Figure S1-5.jpg]

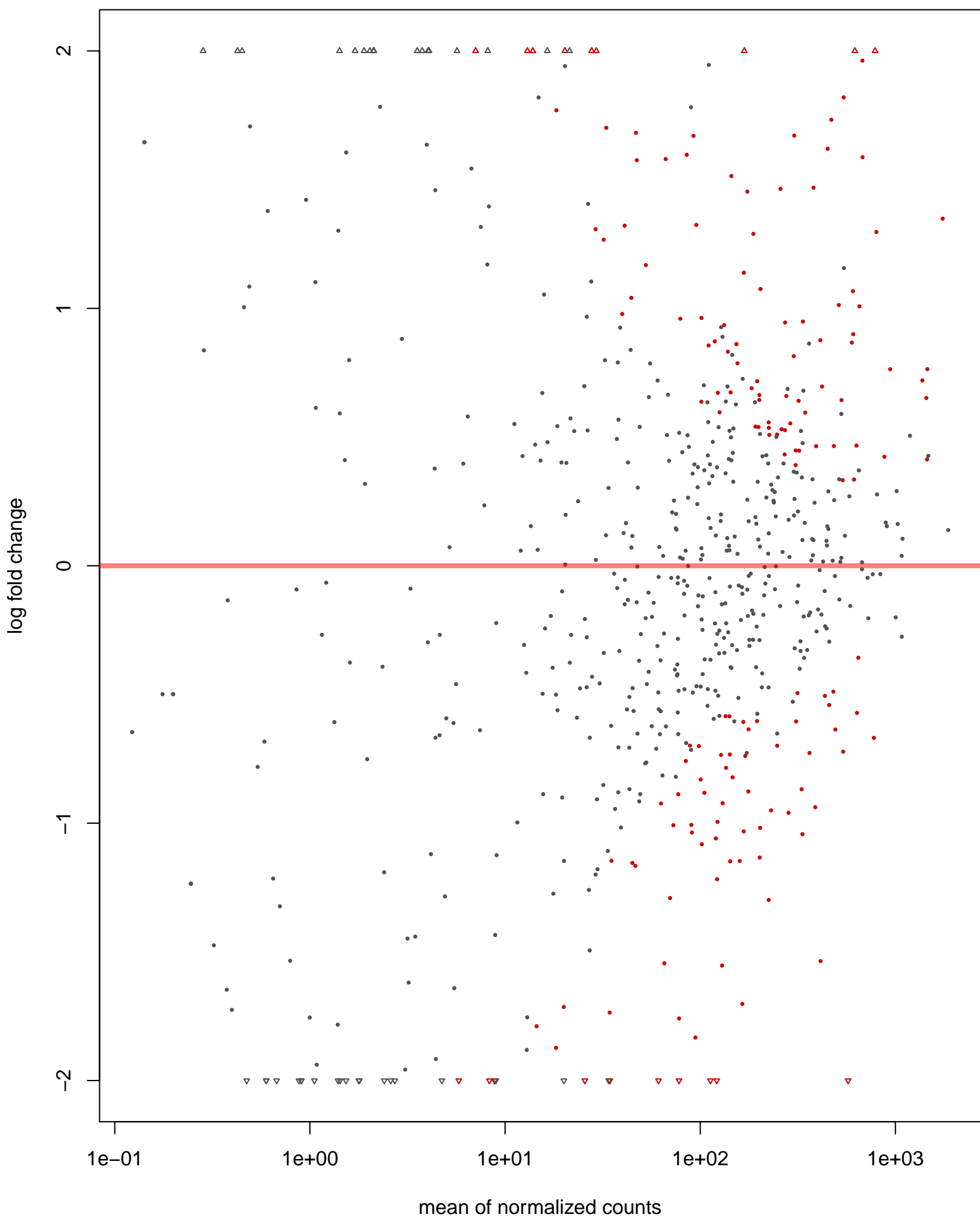

Supplement: Supplementary file 2 — Figure S1. Exon/intron organizations of 743 TaWD40s. Solid yellow boxes and black lines indicate exons and introns, respectively. The scale is shown at the bottom of the figure. Figure S2. MA plots of differentially expressed TaWD40s under biotic and abiotic stresses. MA plots were generated with DESeq2 version 1.20.0. Points are highlighted in red when padj is less than 0.05, representing significantly and differentially expressed TaWD40s. Points falling outside of 2 to − 2 log fold are plotted as open triangles pointing either up or down. (a) Cold stress for 2 weeks, (b) Heat stress for 1 h, (c) Heat stress for 6 h, (d) Drought stress for 1 h, (e) Drought stress for 6 h, (f) Drought and heat stresses for 1 h, (g) Drought and heat stresses for 6 h, (h) Infection of powdery mildew pathogen (E09) for 24 h, (i) Infection of powdery mildew pathogen (E09) for 48 h, (j) Infection of powdery E09 for 72 h, (k) Infection of stripe rust pathogen (CYR31) for 24 h, (l) Infection of CYR31 for 48 h, and (m) Infection of CYR31 for 72 h. Figure S3. Standard and dissociation curves of qRT-PCR. (ZIP 9762 kb) [file 12864_2018_5157_MOESM2_ESM.zip › Figure S2(a).pdf]

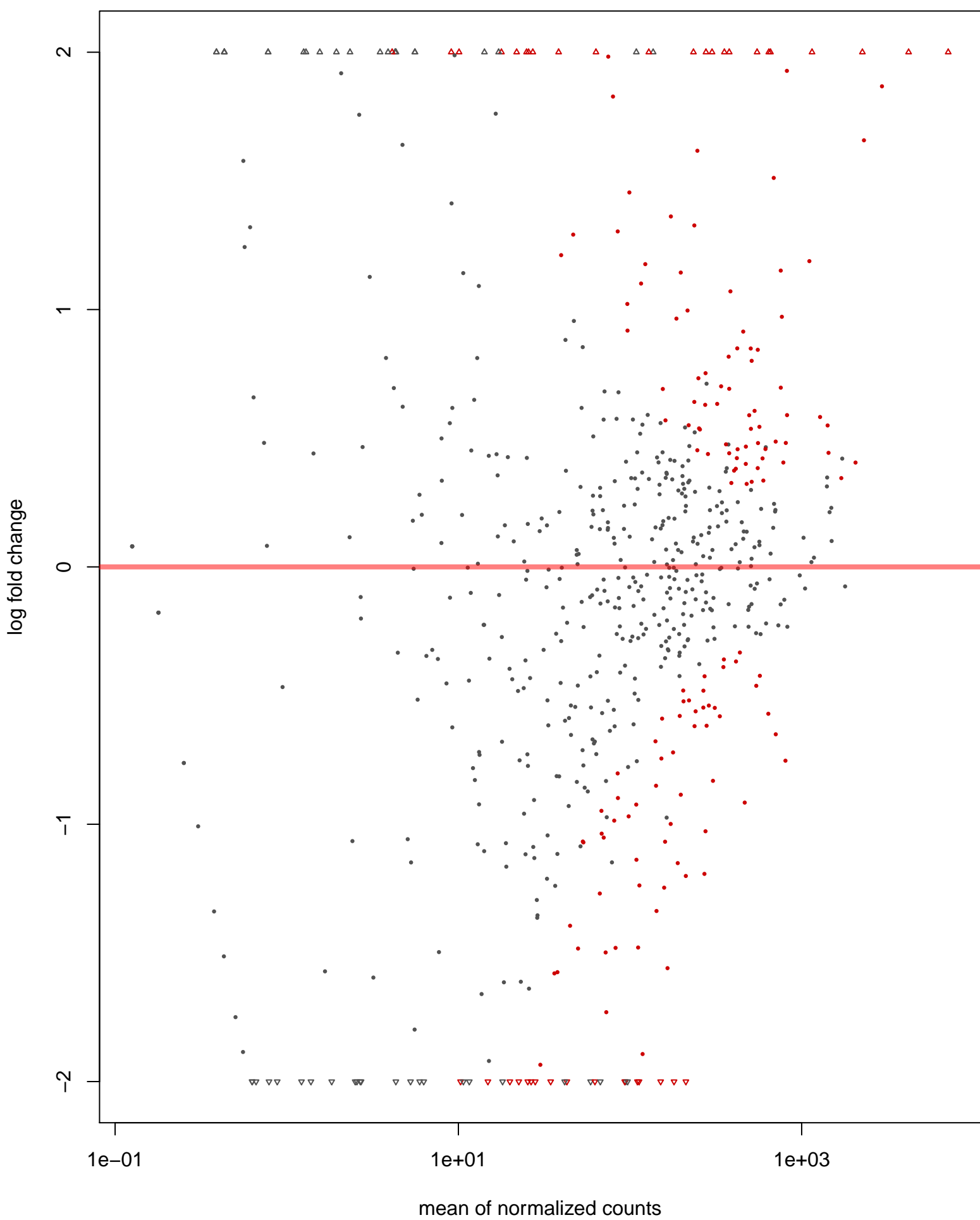

Supplement: Supplementary file 2 — Figure S1. Exon/intron organizations of 743 TaWD40s. Solid yellow boxes and black lines indicate exons and introns, respectively. The scale is shown at the bottom of the figure. Figure S2. MA plots of differentially expressed TaWD40s under biotic and abiotic stresses. MA plots were generated with DESeq2 version 1.20.0. Points are highlighted in red when padj is less than 0.05, representing significantly and differentially expressed TaWD40s. Points falling outside of 2 to − 2 log fold are plotted as open triangles pointing either up or down. (a) Cold stress for 2 weeks, (b) Heat stress for 1 h, (c) Heat stress for 6 h, (d) Drought stress for 1 h, (e) Drought stress for 6 h, (f) Drought and heat stresses for 1 h, (g) Drought and heat stresses for 6 h, (h) Infection of powdery mildew pathogen (E09) for 24 h, (i) Infection of powdery mildew pathogen (E09) for 48 h, (j) Infection of powdery E09 for 72 h, (k) Infection of stripe rust pathogen (CYR31) for 24 h, (l) Infection of CYR31 for 48 h, and (m) Infection of CYR31 for 72 h. Figure S3. Standard and dissociation curves of qRT-PCR. (ZIP 9762 kb) [file 12864_2018_5157_MOESM2_ESM.zip › Figure S2(b).pdf]

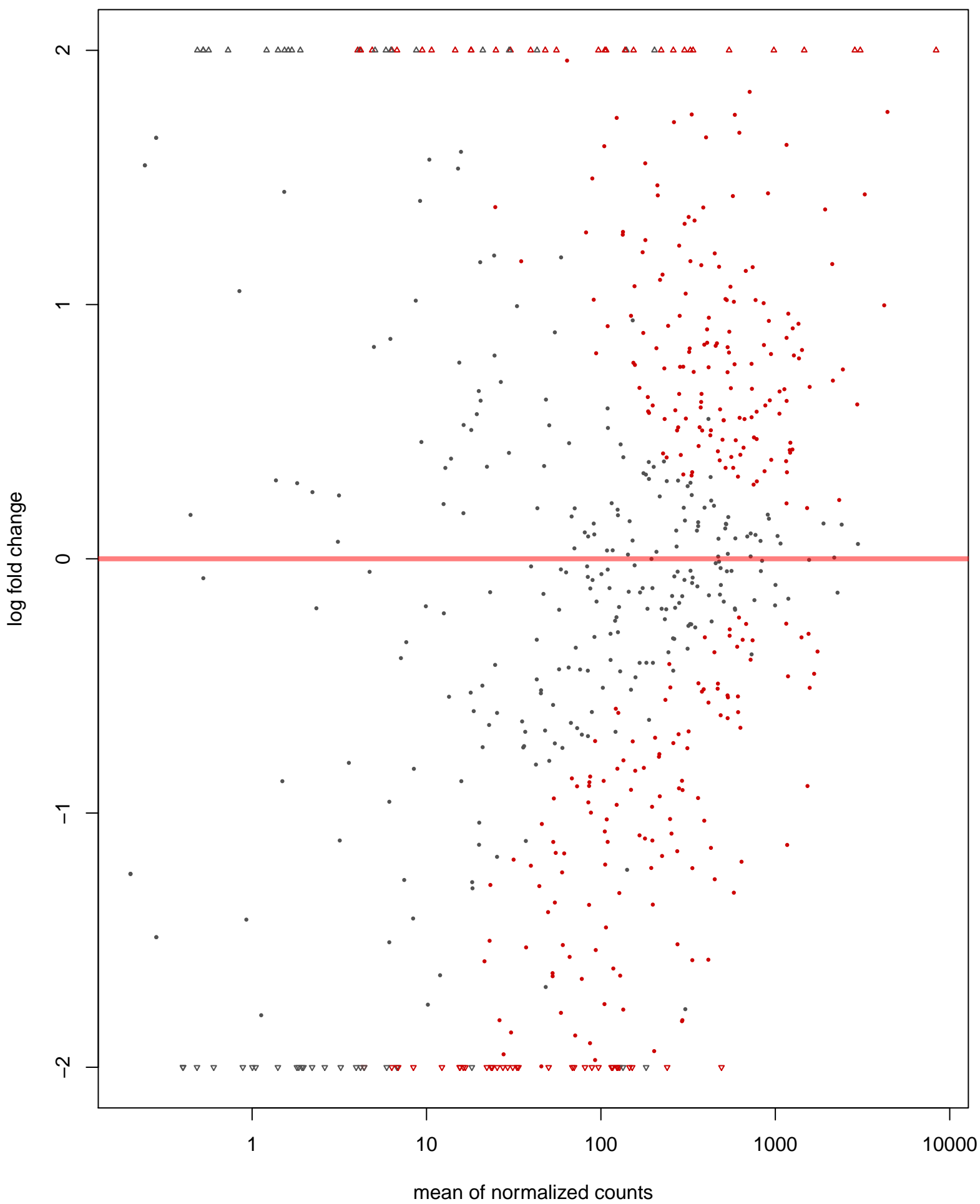

Supplement: Supplementary file 2 — Figure S1. Exon/intron organizations of 743 TaWD40s. Solid yellow boxes and black lines indicate exons and introns, respectively. The scale is shown at the bottom of the figure. Figure S2. MA plots of differentially expressed TaWD40s under biotic and abiotic stresses. MA plots were generated with DESeq2 version 1.20.0. Points are highlighted in red when padj is less than 0.05, representing significantly and differentially expressed TaWD40s. Points falling outside of 2 to − 2 log fold are plotted as open triangles pointing either up or down. (a) Cold stress for 2 weeks, (b) Heat stress for 1 h, (c) Heat stress for 6 h, (d) Drought stress for 1 h, (e) Drought stress for 6 h, (f) Drought and heat stresses for 1 h, (g) Drought and heat stresses for 6 h, (h) Infection of powdery mildew pathogen (E09) for 24 h, (i) Infection of powdery mildew pathogen (E09) for 48 h, (j) Infection of powdery E09 for 72 h, (k) Infection of stripe rust pathogen (CYR31) for 24 h, (l) Infection of CYR31 for 48 h, and (m) Infection of CYR31 for 72 h. Figure S3. Standard and dissociation curves of qRT-PCR. (ZIP 9762 kb) [file 12864_2018_5157_MOESM2_ESM.zip › Figure S2(c).pdf]

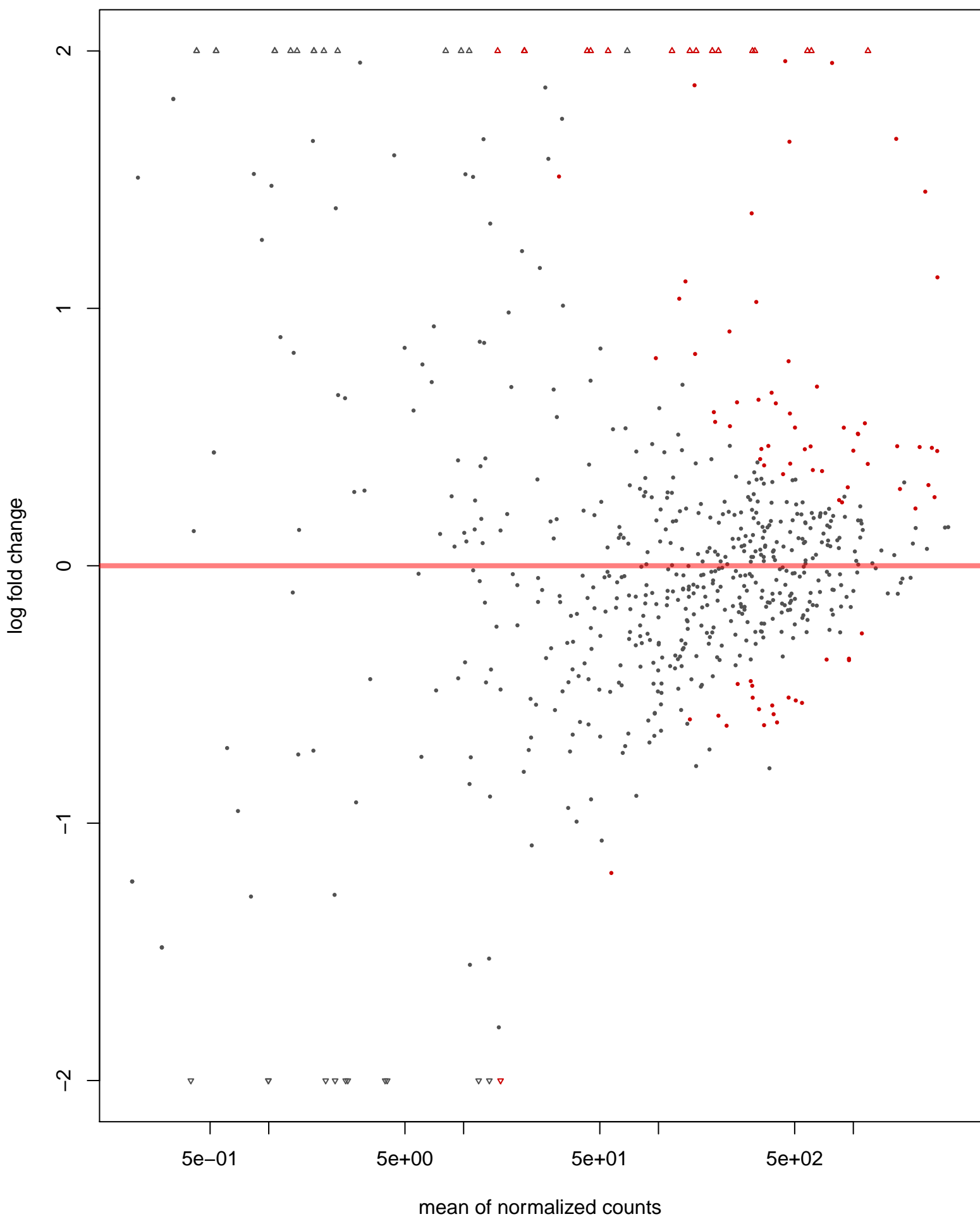

Supplement: Supplementary file 2 — Figure S1. Exon/intron organizations of 743 TaWD40s. Solid yellow boxes and black lines indicate exons and introns, respectively. The scale is shown at the bottom of the figure. Figure S2. MA plots of differentially expressed TaWD40s under biotic and abiotic stresses. MA plots were generated with DESeq2 version 1.20.0. Points are highlighted in red when padj is less than 0.05, representing significantly and differentially expressed TaWD40s. Points falling outside of 2 to − 2 log fold are plotted as open triangles pointing either up or down. (a) Cold stress for 2 weeks, (b) Heat stress for 1 h, (c) Heat stress for 6 h, (d) Drought stress for 1 h, (e) Drought stress for 6 h, (f) Drought and heat stresses for 1 h, (g) Drought and heat stresses for 6 h, (h) Infection of powdery mildew pathogen (E09) for 24 h, (i) Infection of powdery mildew pathogen (E09) for 48 h, (j) Infection of powdery E09 for 72 h, (k) Infection of stripe rust pathogen (CYR31) for 24 h, (l) Infection of CYR31 for 48 h, and (m) Infection of CYR31 for 72 h. Figure S3. Standard and dissociation curves of qRT-PCR. (ZIP 9762 kb) [file 12864_2018_5157_MOESM2_ESM.zip › Figure S2(d).pdf]

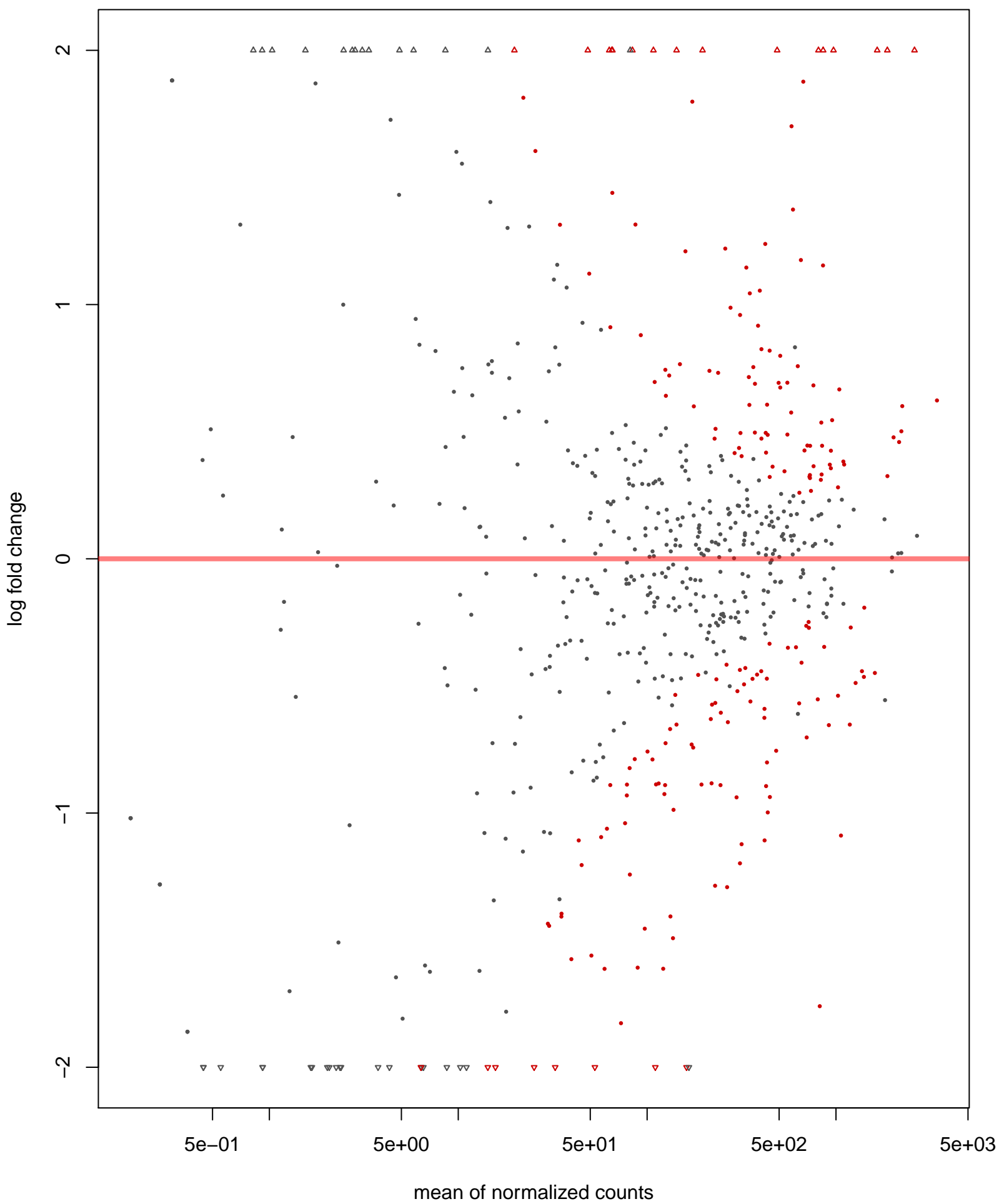

Supplement: Supplementary file 2 — Figure S1. Exon/intron organizations of 743 TaWD40s. Solid yellow boxes and black lines indicate exons and introns, respectively. The scale is shown at the bottom of the figure. Figure S2. MA plots of differentially expressed TaWD40s under biotic and abiotic stresses. MA plots were generated with DESeq2 version 1.20.0. Points are highlighted in red when padj is less than 0.05, representing significantly and differentially expressed TaWD40s. Points falling outside of 2 to − 2 log fold are plotted as open triangles pointing either up or down. (a) Cold stress for 2 weeks, (b) Heat stress for 1 h, (c) Heat stress for 6 h, (d) Drought stress for 1 h, (e) Drought stress for 6 h, (f) Drought and heat stresses for 1 h, (g) Drought and heat stresses for 6 h, (h) Infection of powdery mildew pathogen (E09) for 24 h, (i) Infection of powdery mildew pathogen (E09) for 48 h, (j) Infection of powdery E09 for 72 h, (k) Infection of stripe rust pathogen (CYR31) for 24 h, (l) Infection of CYR31 for 48 h, and (m) Infection of CYR31 for 72 h. Figure S3. Standard and dissociation curves of qRT-PCR. (ZIP 9762 kb) [file 12864_2018_5157_MOESM2_ESM.zip › Figure S2(e).pdf]

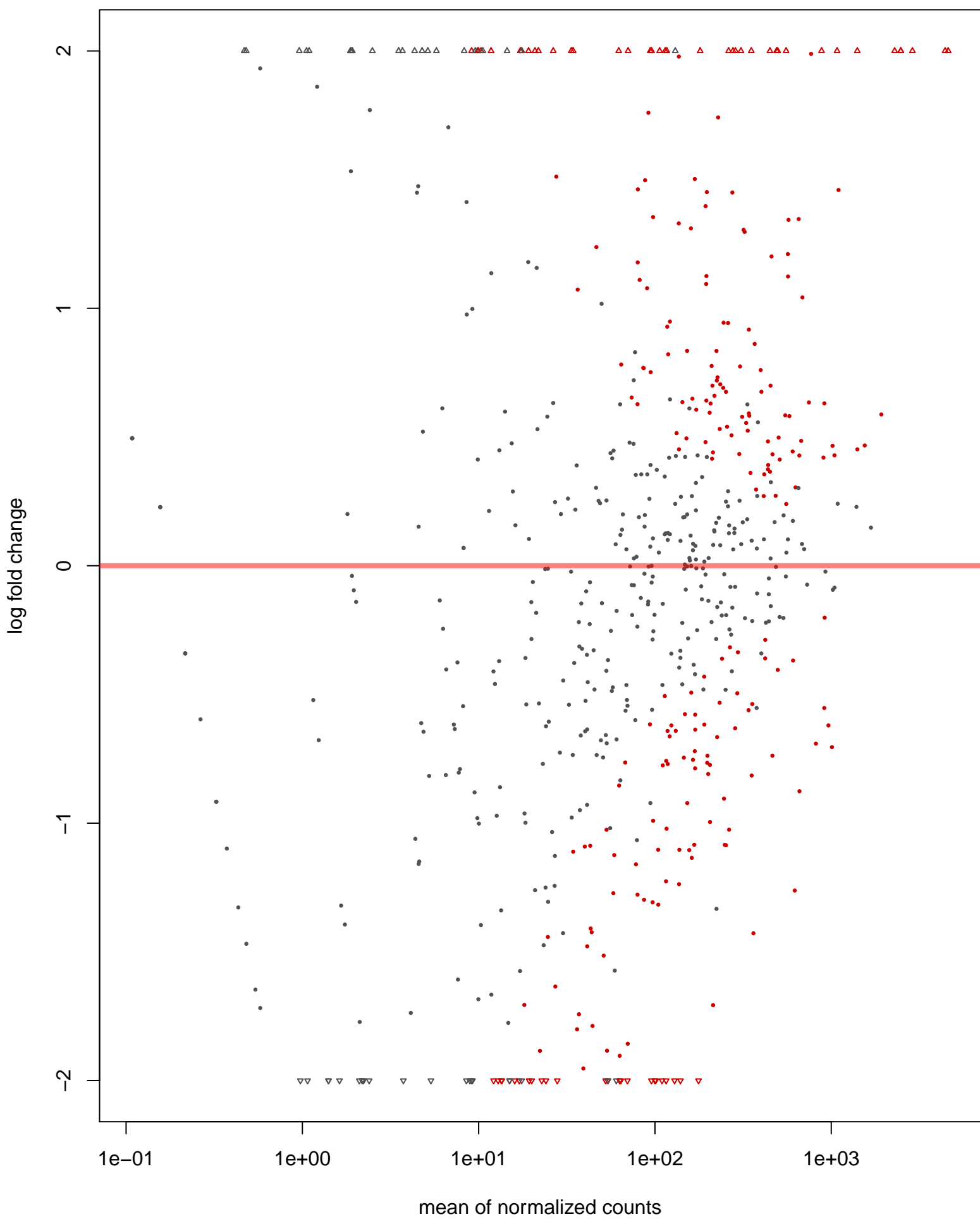

Supplement: Supplementary file 2 — Figure S1. Exon/intron organizations of 743 TaWD40s. Solid yellow boxes and black lines indicate exons and introns, respectively. The scale is shown at the bottom of the figure. Figure S2. MA plots of differentially expressed TaWD40s under biotic and abiotic stresses. MA plots were generated with DESeq2 version 1.20.0. Points are highlighted in red when padj is less than 0.05, representing significantly and differentially expressed TaWD40s. Points falling outside of 2 to − 2 log fold are plotted as open triangles pointing either up or down. (a) Cold stress for 2 weeks, (b) Heat stress for 1 h, (c) Heat stress for 6 h, (d) Drought stress for 1 h, (e) Drought stress for 6 h, (f) Drought and heat stresses for 1 h, (g) Drought and heat stresses for 6 h, (h) Infection of powdery mildew pathogen (E09) for 24 h, (i) Infection of powdery mildew pathogen (E09) for 48 h, (j) Infection of powdery E09 for 72 h, (k) Infection of stripe rust pathogen (CYR31) for 24 h, (l) Infection of CYR31 for 48 h, and (m) Infection of CYR31 for 72 h. Figure S3. Standard and dissociation curves of qRT-PCR. (ZIP 9762 kb) [file 12864_2018_5157_MOESM2_ESM.zip › Figure S2(f).pdf]

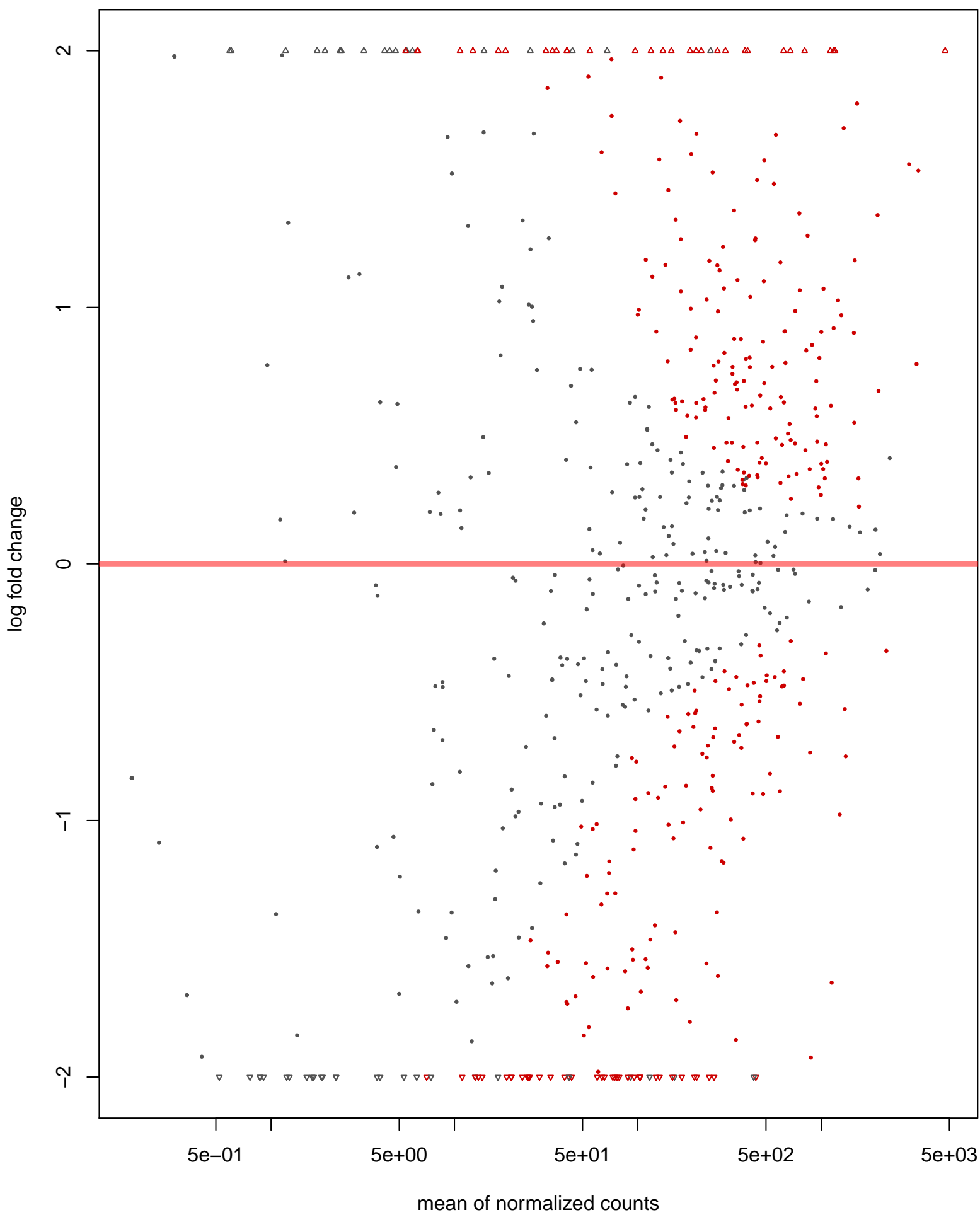

Supplement: Supplementary file 2 — Figure S1. Exon/intron organizations of 743 TaWD40s. Solid yellow boxes and black lines indicate exons and introns, respectively. The scale is shown at the bottom of the figure. Figure S2. MA plots of differentially expressed TaWD40s under biotic and abiotic stresses. MA plots were generated with DESeq2 version 1.20.0. Points are highlighted in red when padj is less than 0.05, representing significantly and differentially expressed TaWD40s. Points falling outside of 2 to − 2 log fold are plotted as open triangles pointing either up or down. (a) Cold stress for 2 weeks, (b) Heat stress for 1 h, (c) Heat stress for 6 h, (d) Drought stress for 1 h, (e) Drought stress for 6 h, (f) Drought and heat stresses for 1 h, (g) Drought and heat stresses for 6 h, (h) Infection of powdery mildew pathogen (E09) for 24 h, (i) Infection of powdery mildew pathogen (E09) for 48 h, (j) Infection of powdery E09 for 72 h, (k) Infection of stripe rust pathogen (CYR31) for 24 h, (l) Infection of CYR31 for 48 h, and (m) Infection of CYR31 for 72 h. Figure S3. Standard and dissociation curves of qRT-PCR. (ZIP 9762 kb) [file 12864_2018_5157_MOESM2_ESM.zip › Figure S2(g).pdf]

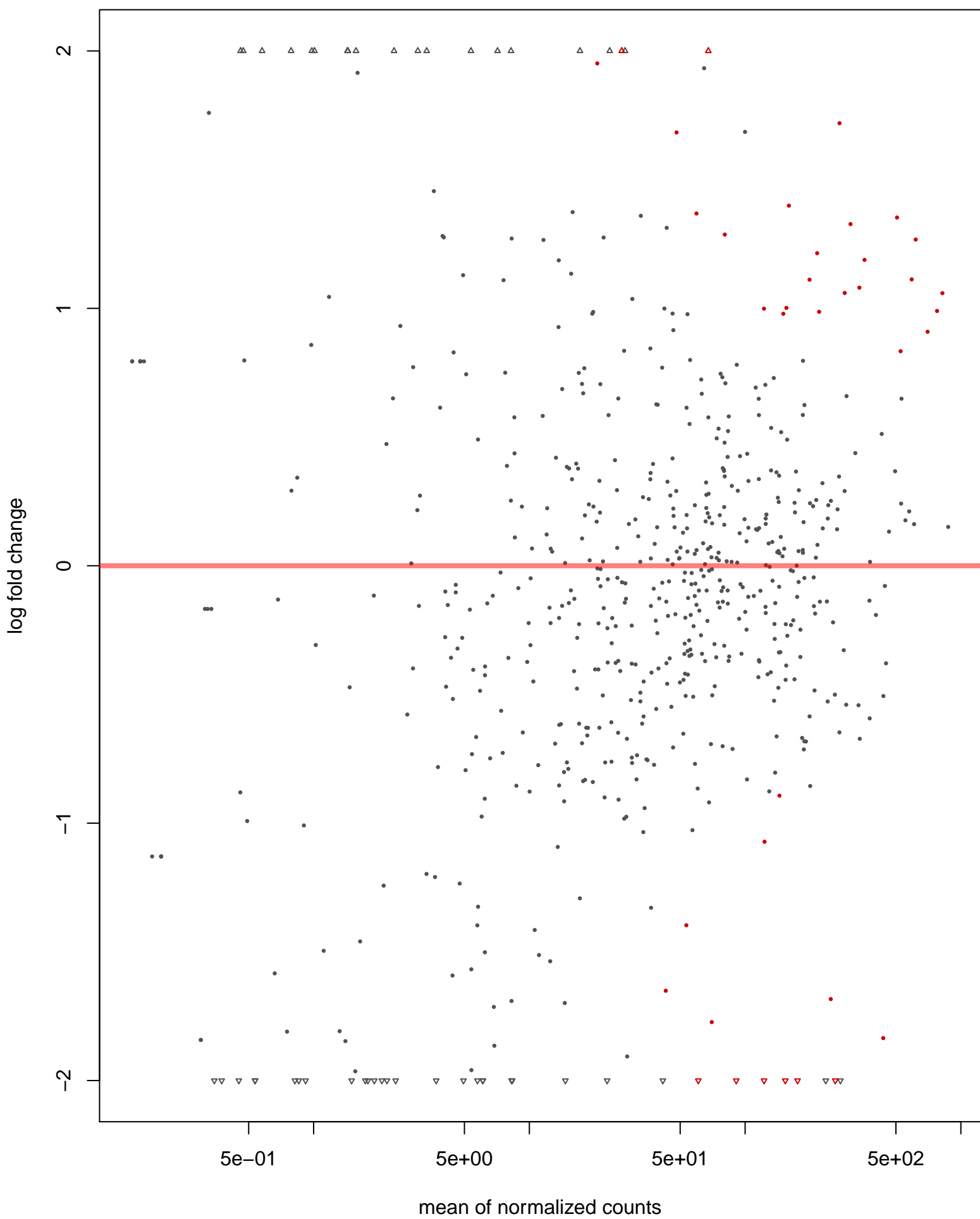

Supplement: Supplementary file 2 — Figure S1. Exon/intron organizations of 743 TaWD40s. Solid yellow boxes and black lines indicate exons and introns, respectively. The scale is shown at the bottom of the figure. Figure S2. MA plots of differentially expressed TaWD40s under biotic and abiotic stresses. MA plots were generated with DESeq2 version 1.20.0. Points are highlighted in red when padj is less than 0.05, representing significantly and differentially expressed TaWD40s. Points falling outside of 2 to − 2 log fold are plotted as open triangles pointing either up or down. (a) Cold stress for 2 weeks, (b) Heat stress for 1 h, (c) Heat stress for 6 h, (d) Drought stress for 1 h, (e) Drought stress for 6 h, (f) Drought and heat stresses for 1 h, (g) Drought and heat stresses for 6 h, (h) Infection of powdery mildew pathogen (E09) for 24 h, (i) Infection of powdery mildew pathogen (E09) for 48 h, (j) Infection of powdery E09 for 72 h, (k) Infection of stripe rust pathogen (CYR31) for 24 h, (l) Infection of CYR31 for 48 h, and (m) Infection of CYR31 for 72 h. Figure S3. Standard and dissociation curves of qRT-PCR. (ZIP 9762 kb) [file 12864_2018_5157_MOESM2_ESM.zip › Figure S2(h).pdf]

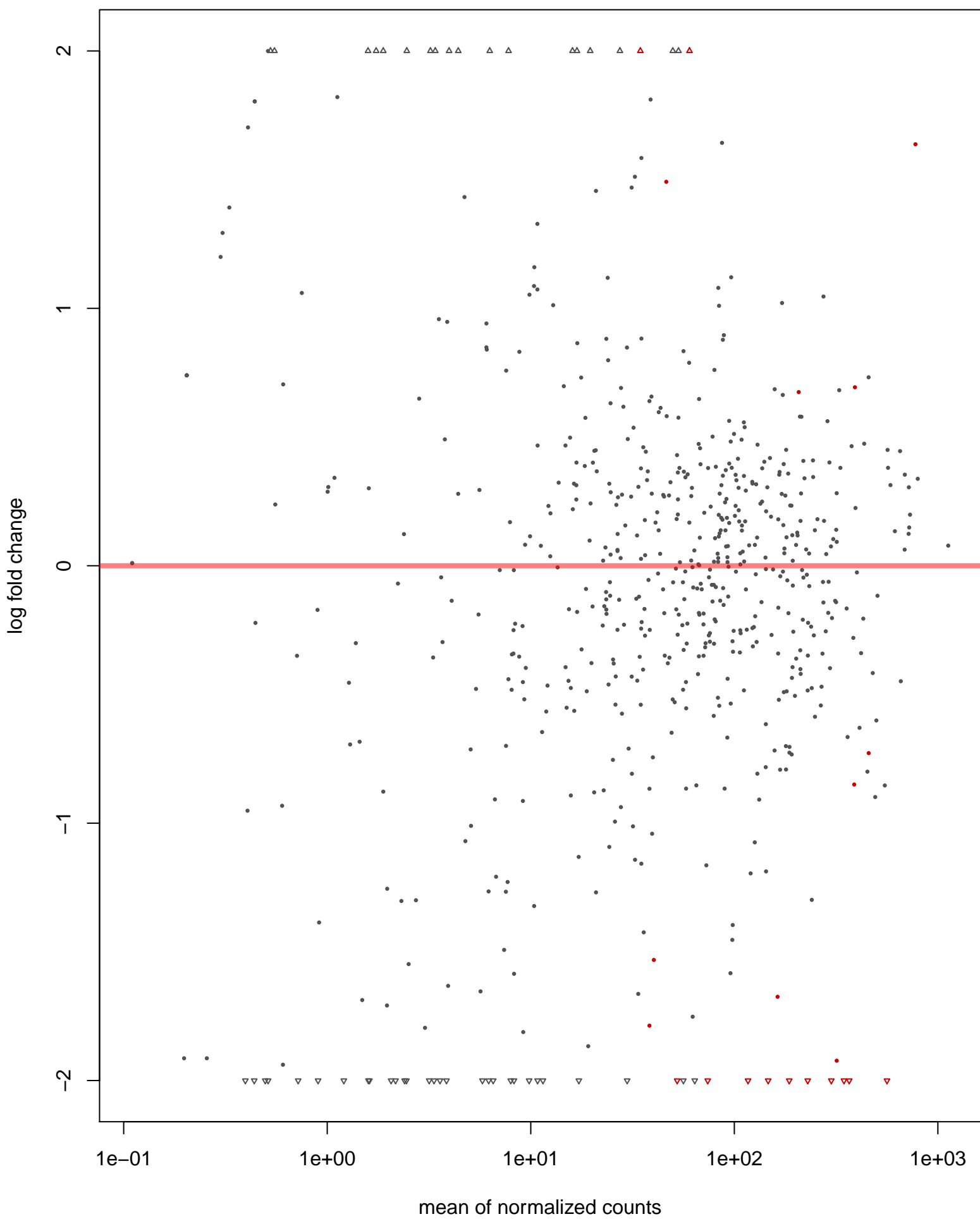

Supplement: Supplementary file 2 — Figure S1. Exon/intron organizations of 743 TaWD40s. Solid yellow boxes and black lines indicate exons and introns, respectively. The scale is shown at the bottom of the figure. Figure S2. MA plots of differentially expressed TaWD40s under biotic and abiotic stresses. MA plots were generated with DESeq2 version 1.20.0. Points are highlighted in red when padj is less than 0.05, representing significantly and differentially expressed TaWD40s. Points falling outside of 2 to − 2 log fold are plotted as open triangles pointing either up or down. (a) Cold stress for 2 weeks, (b) Heat stress for 1 h, (c) Heat stress for 6 h, (d) Drought stress for 1 h, (e) Drought stress for 6 h, (f) Drought and heat stresses for 1 h, (g) Drought and heat stresses for 6 h, (h) Infection of powdery mildew pathogen (E09) for 24 h, (i) Infection of powdery mildew pathogen (E09) for 48 h, (j) Infection of powdery E09 for 72 h, (k) Infection of stripe rust pathogen (CYR31) for 24 h, (l) Infection of CYR31 for 48 h, and (m) Infection of CYR31 for 72 h. Figure S3. Standard and dissociation curves of qRT-PCR. (ZIP 9762 kb) [file 12864_2018_5157_MOESM2_ESM.zip › Figure S2(i).pdf]

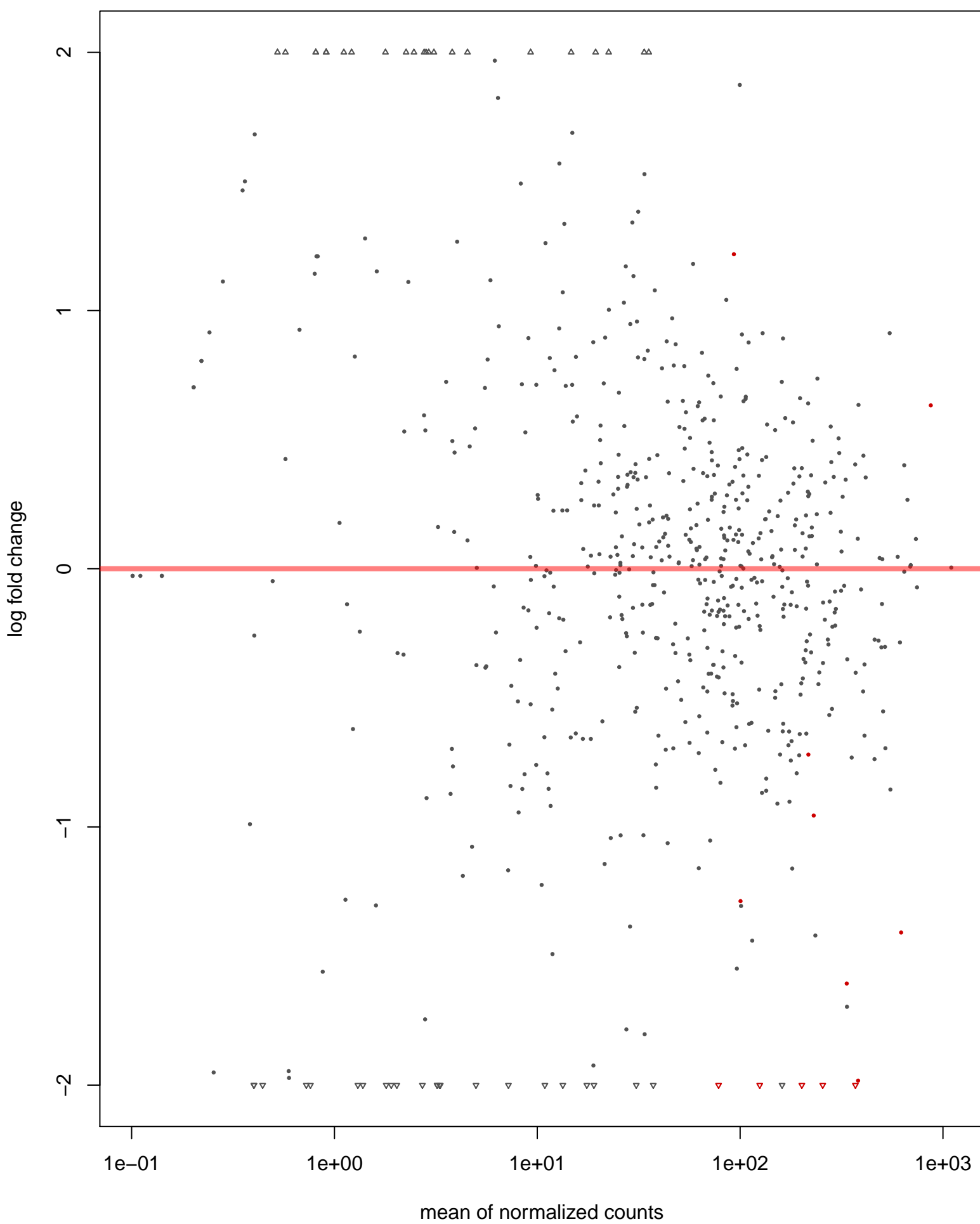

Supplement: Supplementary file 2 — Figure S1. Exon/intron organizations of 743 TaWD40s. Solid yellow boxes and black lines indicate exons and introns, respectively. The scale is shown at the bottom of the figure. Figure S2. MA plots of differentially expressed TaWD40s under biotic and abiotic stresses. MA plots were generated with DESeq2 version 1.20.0. Points are highlighted in red when padj is less than 0.05, representing significantly and differentially expressed TaWD40s. Points falling outside of 2 to − 2 log fold are plotted as open triangles pointing either up or down. (a) Cold stress for 2 weeks, (b) Heat stress for 1 h, (c) Heat stress for 6 h, (d) Drought stress for 1 h, (e) Drought stress for 6 h, (f) Drought and heat stresses for 1 h, (g) Drought and heat stresses for 6 h, (h) Infection of powdery mildew pathogen (E09) for 24 h, (i) Infection of powdery mildew pathogen (E09) for 48 h, (j) Infection of powdery E09 for 72 h, (k) Infection of stripe rust pathogen (CYR31) for 24 h, (l) Infection of CYR31 for 48 h, and (m) Infection of CYR31 for 72 h. Figure S3. Standard and dissociation curves of qRT-PCR. (ZIP 9762 kb) [file 12864_2018_5157_MOESM2_ESM.zip › Figure S2(j).pdf]

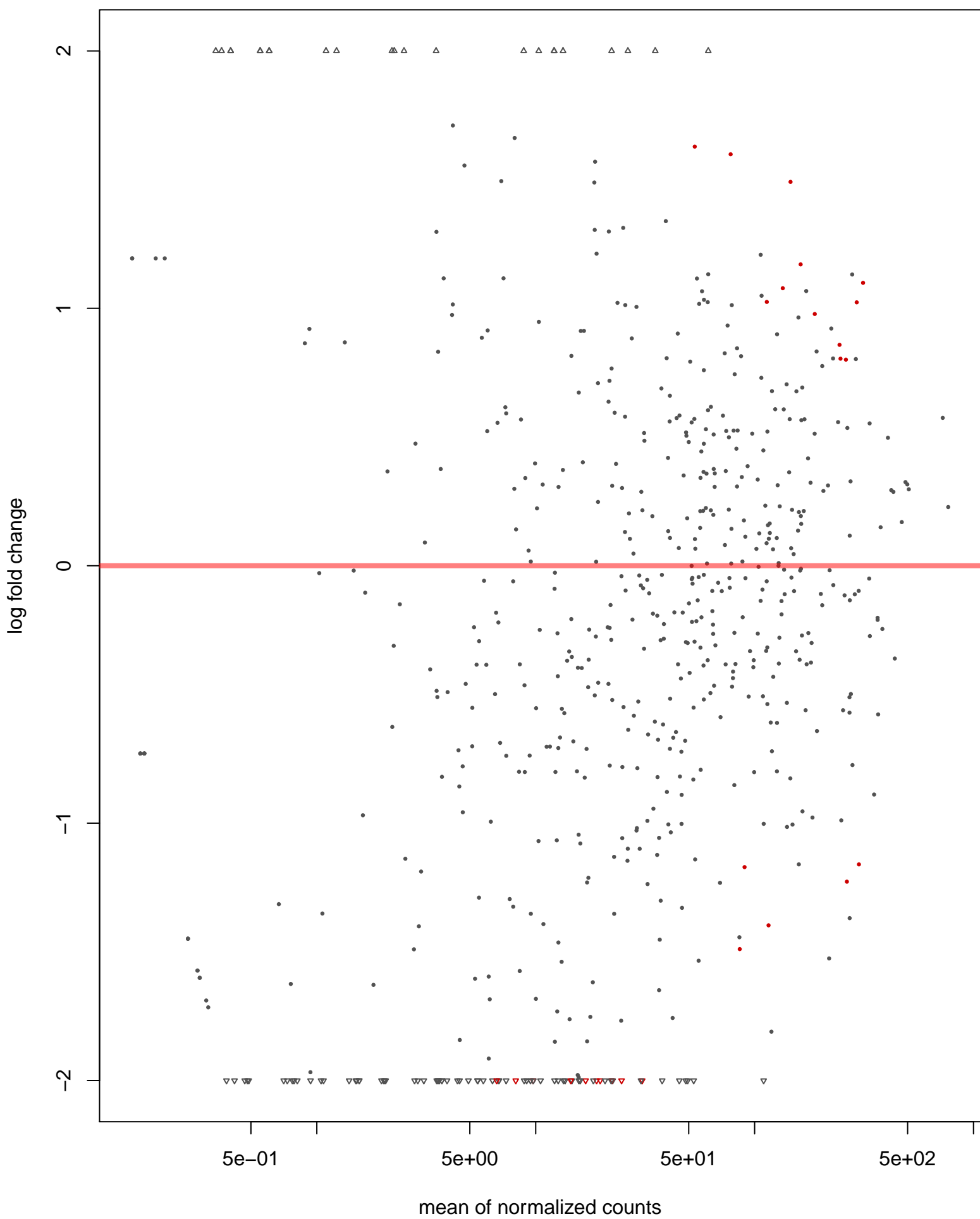

Supplement: Supplementary file 2 — Figure S1. Exon/intron organizations of 743 TaWD40s. Solid yellow boxes and black lines indicate exons and introns, respectively. The scale is shown at the bottom of the figure. Figure S2. MA plots of differentially expressed TaWD40s under biotic and abiotic stresses. MA plots were generated with DESeq2 version 1.20.0. Points are highlighted in red when padj is less than 0.05, representing significantly and differentially expressed TaWD40s. Points falling outside of 2 to − 2 log fold are plotted as open triangles pointing either up or down. (a) Cold stress for 2 weeks, (b) Heat stress for 1 h, (c) Heat stress for 6 h, (d) Drought stress for 1 h, (e) Drought stress for 6 h, (f) Drought and heat stresses for 1 h, (g) Drought and heat stresses for 6 h, (h) Infection of powdery mildew pathogen (E09) for 24 h, (i) Infection of powdery mildew pathogen (E09) for 48 h, (j) Infection of powdery E09 for 72 h, (k) Infection of stripe rust pathogen (CYR31) for 24 h, (l) Infection of CYR31 for 48 h, and (m) Infection of CYR31 for 72 h. Figure S3. Standard and dissociation curves of qRT-PCR. (ZIP 9762 kb) [file 12864_2018_5157_MOESM2_ESM.zip › Figure S2(k).pdf]

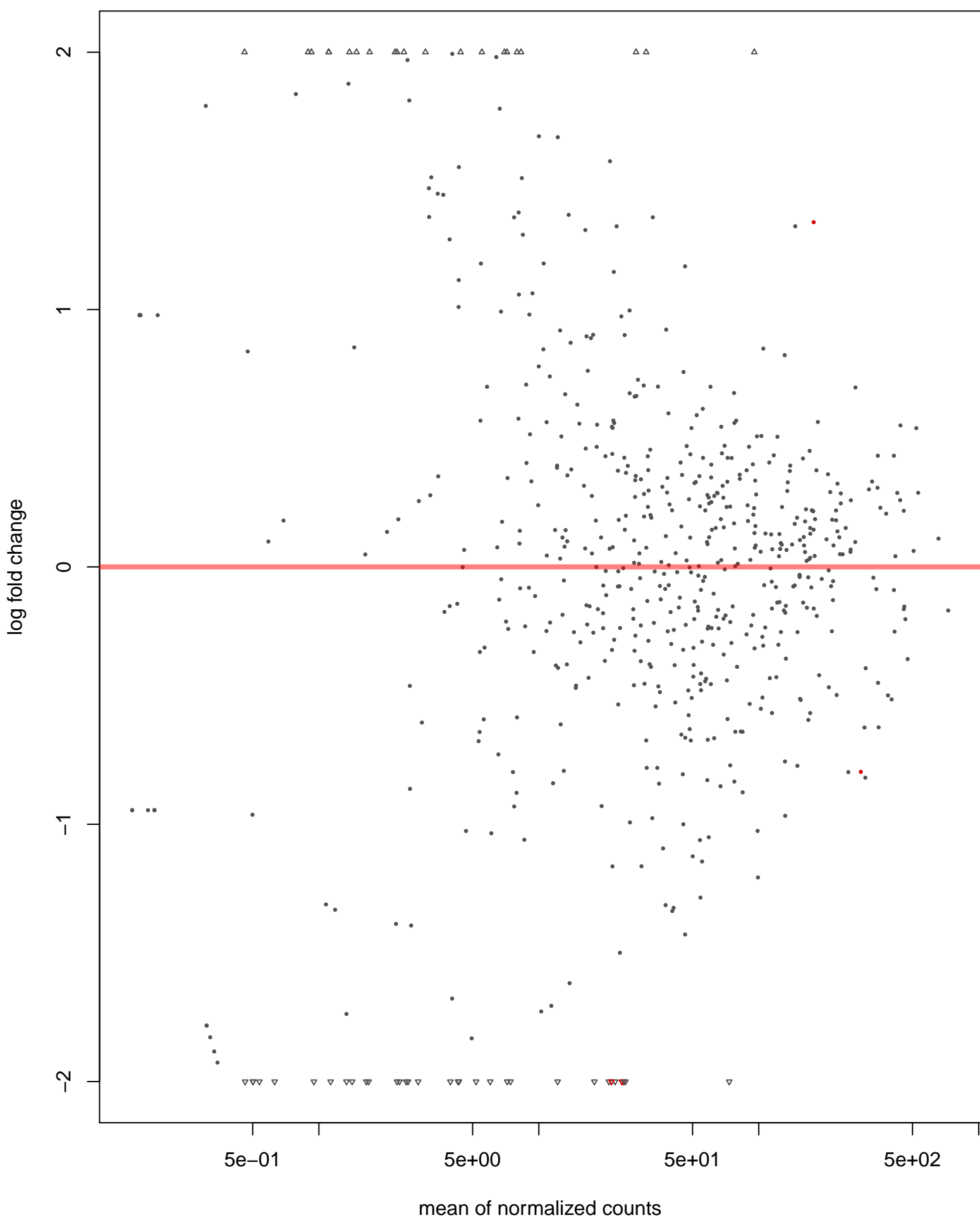

Supplement: Supplementary file 2 — Figure S1. Exon/intron organizations of 743 TaWD40s. Solid yellow boxes and black lines indicate exons and introns, respectively. The scale is shown at the bottom of the figure. Figure S2. MA plots of differentially expressed TaWD40s under biotic and abiotic stresses. MA plots were generated with DESeq2 version 1.20.0. Points are highlighted in red when padj is less than 0.05, representing significantly and differentially expressed TaWD40s. Points falling outside of 2 to − 2 log fold are plotted as open triangles pointing either up or down. (a) Cold stress for 2 weeks, (b) Heat stress for 1 h, (c) Heat stress for 6 h, (d) Drought stress for 1 h, (e) Drought stress for 6 h, (f) Drought and heat stresses for 1 h, (g) Drought and heat stresses for 6 h, (h) Infection of powdery mildew pathogen (E09) for 24 h, (i) Infection of powdery mildew pathogen (E09) for 48 h, (j) Infection of powdery E09 for 72 h, (k) Infection of stripe rust pathogen (CYR31) for 24 h, (l) Infection of CYR31 for 48 h, and (m) Infection of CYR31 for 72 h. Figure S3. Standard and dissociation curves of qRT-PCR. (ZIP 9762 kb) [file 12864_2018_5157_MOESM2_ESM.zip › Figure S2(l).pdf]

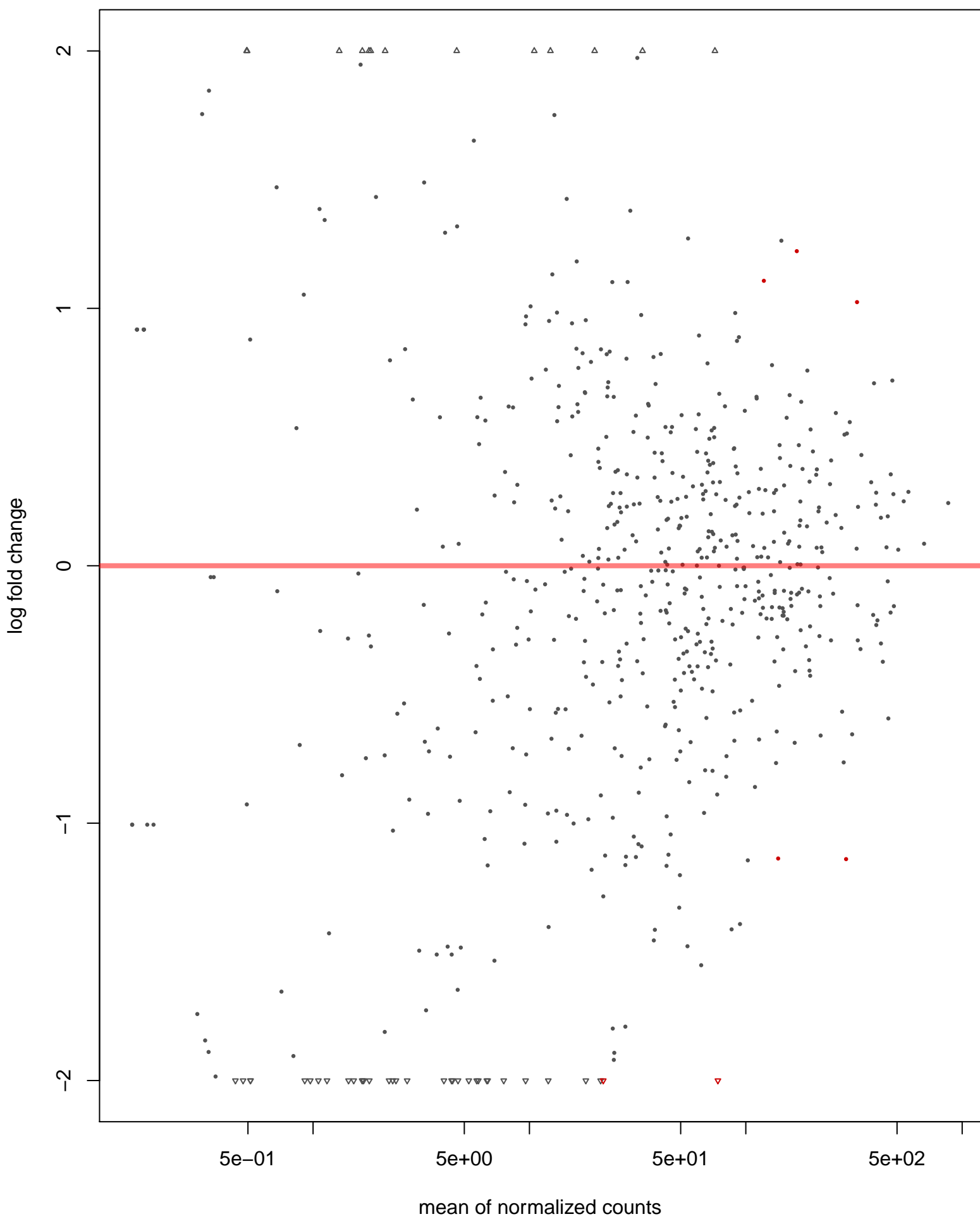

Supplement: Supplementary file 2 — Figure S1. Exon/intron organizations of 743 TaWD40s. Solid yellow boxes and black lines indicate exons and introns, respectively. The scale is shown at the bottom of the figure. Figure S2. MA plots of differentially expressed TaWD40s under biotic and abiotic stresses. MA plots were generated with DESeq2 version 1.20.0. Points are highlighted in red when padj is less than 0.05, representing significantly and differentially expressed TaWD40s. Points falling outside of 2 to − 2 log fold are plotted as open triangles pointing either up or down. (a) Cold stress for 2 weeks, (b) Heat stress for 1 h, (c) Heat stress for 6 h, (d) Drought stress for 1 h, (e) Drought stress for 6 h, (f) Drought and heat stresses for 1 h, (g) Drought and heat stresses for 6 h, (h) Infection of powdery mildew pathogen (E09) for 24 h, (i) Infection of powdery mildew pathogen (E09) for 48 h, (j) Infection of powdery E09 for 72 h, (k) Infection of stripe rust pathogen (CYR31) for 24 h, (l) Infection of CYR31 for 48 h, and (m) Infection of CYR31 for 72 h. Figure S3. Standard and dissociation curves of qRT-PCR. (ZIP 9762 kb) [file 12864_2018_5157_MOESM2_ESM.zip › Figure S2(m).pdf]
